# Stem Cell-Derived Extracellular Vesicles for the Treatment of Osteoporosis: A Systematic Review of Preclinical Evidence

**DOI:** 10.3390/biomedicines14091976

**Published:** 2026-09-02

**Authors:** Yi Yang, Lifen Qiao, Yan Huang, Qin Xia, Hui Xie

**Affiliations:** 1 Department of Geriatrics, Tongji Hospital, Tongji Medical College, Huazhong University of Science and Technology, Wuhan 430030, China; yangyi@tjh.tjmu.edu.cn (Y.Y.); 164101898@qq.com (L.Q.); 2 Department of Rehabilitation Medicine, Tongji Hospital, Tongji Medical College, Huazhong University of Science and Technology, Wuhan 430030, China; baobaoxiong0401@163.com

**Keywords:** stem cells, extracellular vesicles, osteoporosis

## Abstract

**Background**: Osteoporosis is a prevalent skeletal disorder characterized by reduced bone mass and microarchitectural deterioration, resulting in an increased risk of fractures. Stem cell-derived extracellular vesicles (EVs) have emerged as a promising cell-free therapeutic strategy owing to their inherent advantages, including excellent biocompatibility, low immunogenicity, diverse biological activities, and engineering potential. Despite growing interest in this field, the existing preclinical evidence remains highly heterogeneous in terms of study design, stem cell sources, EV engineering strategies, and delivery approaches. This systematic review aimed to comprehensively synthesize and critically evaluate the available preclinical evidence regarding the therapeutic potential of stem cell-derived EVs for osteoporosis. **Methods**: A systematic literature search was conducted in PubMed, Embase, and Web of Science in accordance with the Preferred Reporting Items for Systematic Reviews and Meta-Analyses (PRISMA) guidelines. Controlled animal studies investigating the therapeutic effects of stem cell-derived EVs in osteoporosis models were included. Data on stem cell sources, EV characteristics, engineering approaches, administration strategies, osteoporosis animal models, therapeutic outcomes, and underlying mechanisms were extracted and qualitatively synthesized. **Results**: A total of 64 studies were included. Overall, stem cell-derived EVs demonstrated promising therapeutic potential across various animal models of osteoporosis, exerting pleiotropic effects, including promotion of osteogenesis, inhibition of osteoclastogenesis, enhancement of angiogenesis, modulation of immune responses, and regulation of cellular behaviors. Mechanistically, these effects were largely attributed to the transfer of diverse bioactive cargo, including nucleic acids, proteins, and lipids, which collectively regulate multiple signaling pathways involved in bone remodeling. **Conclusions**: Current preclinical evidence supports stem cell-derived EVs as a promising cell-free therapeutic strategy for osteoporosis. However, variability in study design and incomplete reporting in primary studies limit cross-study comparability and translational interpretation, underscoring the need for improved methodological rigor, standardized reporting practices, and robust large-animal validation studies to facilitate future clinical translation.

## 1. Introduction

Osteoporosis is a systemic metabolic bone disorder characterized by reduced bone mass, progressive deterioration of bone microarchitecture, and compromised bone strength. The fundamental pathological basis of osteoporosis lies in the disruption of the dynamic balance between bone formation and bone resorption [1]. With the continued aging of the global population, the prevalence of osteoporosis is steadily increasing, making it a major contributor to disability and morbidity among the elderly. Epidemiological evidence indicates that osteoporotic fractures, particularly those affecting the hip and vertebrae, not only significantly impair patients’ quality of life but are also strongly associated with increased mortality, thereby imposing a substantial burden on healthcare systems worldwide [2].

Bone remodeling is a highly coordinated process driven by the coupled activities of osteoblasts and osteoclasts and tightly regulated by a complex regulatory network involving systemic hormones, local microenvironmental factors, mechanical stimuli, immune and inflammatory responses, and metabolic status. Under osteoporotic conditions, this finely balanced homeostasis is disrupted, resulting in impaired osteogenesis and increased bone resorption by osteoclasts [3,4,5,6,7]. Current clinical management of osteoporosis primarily relies on antiresorptive agents, such as bisphosphonates and RANKL-targeted inhibitors, as well as anabolic therapies, including parathyroid hormone analogs and sclerostin inhibitors [8]. Although these interventions have demonstrated efficacy in increasing bone mineral density (BMD) and reducing fracture risk, they are associated with several limitations, including concerns regarding long-term safety, rebound bone loss following treatment discontinuation, and a limited ability to fully restore bone microarchitecture [9]. Therefore, the development of novel therapeutic strategies that are safer, more effective, and capable of promoting functional bone regeneration remains of considerable clinical importance.

In recent decades, stem cells, particularly mesenchymal stem cells (MSCs), have been extensively investigated in bone tissue engineering and regenerative medicine owing to their multilineage differentiation capacity, pro-angiogenic potential, and immunomodulatory properties [10]. However, accumulating evidence indicates that the therapeutic effects of MSCs in tissue repair are largely attributed to paracrine mechanisms that modulate the local microenvironment, rather than direct differentiation into parenchymal cells to restore or replace damaged tissue [11]. This evolving understanding has driven a paradigm shift from a “cell replacement” strategy toward approaches centered on cell-derived bioactive factors and their regulatory functions.

Extracellular vesicles (EVs), as key mediators of paracrine signaling, have attracted increasing attention in recent years. EVs comprise a heterogeneous population of lipid bilayer-enclosed vesicles secreted by cells [12]. Traditionally, they have been classified into three main subtypes based on their biogenesis: exosomes, which originate from the inward budding of the endosomal membrane; microvesicles (also referred to as microparticles), which are formed through outward budding of the plasma membrane; and apoptotic bodies, which are released during programmed cell death [13]. However, despite differences in their size, origin, and biogenesis, these subtypes exhibit considerable overlap in their physicochemical characteristics. In addition, currently available isolation techniques are still insufficient to reliably distinguish among EV subtypes, and definitive molecular markers for their unambiguous classification remain lacking. Accordingly, the International Society for Extracellular Vesicles (ISEV) recommends the use of the generic term “EVs” unless specific subtypes have been rigorously isolated and characterized [14]. During their formation, EVs selectively incorporate a diverse array of bioactive molecules derived from parental cells, including nucleic acids, proteins, and lipids. By delivering their cargo to recipient cells via mechanisms such as direct membrane fusion, endocytosis, and ligand-receptor interactions, EVs transmit biological information between cells, thereby regulating cellular functions and playing crucial roles in a broad spectrum of physiological and pathological processes [15].

Compared with their parent cells, stem cell-derived EVs offer several advantages for therapeutic applications, including low immunogenicity, excellent biocompatibility, inherent bioactivity, and amenability to customized engineering [16]. Consequently, EVs have emerged as a promising cell-free therapeutic strategy with strong translational potential. In the context of osteoporosis, accumulating evidence indicates that stem cell-derived EVs regulate bone remodeling through multiple mechanisms. These include promoting the osteogenic differentiation of MSCs and osteolineage cells, inhibiting osteoclastogenesis, modulating the osteoimmune microenvironment, and enhancing angiogenesis, thereby exerting synergistic effects during bone regeneration [17]. Despite growing interest in this field, the available preclinical evidence remains highly heterogeneous with respect to experimental design, stem cell sources, EV subtypes, engineering strategies, and delivery approaches, limiting the ability to comprehensively evaluate the therapeutic potential and translational prospects of stem cell-derived EVs for osteoporosis. Therefore, this systematic review aims to provide an updated and comprehensive synthesis of the current preclinical evidence regarding the application of stem cell-derived EVs for osteoporosis treatment, with particular emphasis on EV sources and subtypes, biological functions, underlying molecular mechanisms, and recent advances in EV engineering strategies for functional modification. In addition, current challenges and translational barriers, including EV preparation, standardization, scalability, and quality control requirements, are critically discussed, and future perspectives are proposed to advance EV-based therapies toward clinical translation.

## 2. Materials and Methods

### 2.1. Systematic Review

This systematic review was conducted following the Preferred Reporting Items for Systematic Reviews and Meta-Analyses (PRISMA) guidelines [18,19]. The study protocol was registered with PROSPERO (CRD420261329281). Study selection, data extraction, and risk of bias assessment were independently performed by two reviewers. Any discrepancies were resolved through discussion and, when necessary, consultation with a third reviewer to reach consensus.

### 2.2. Search Strategies

A comprehensive and systematic literature search was performed in PubMed, Embase, and Web of Science from database inception to 5 March 2026, to identify studies investigating the use of stem cell-derived EVs for the treatment of osteoporosis. The search strategy was developed by one reviewer and reviewed by a second reviewer. Both Medical Subject Headings (MeSH) terms (including “stem cells,” “extracellular vesicles,” “exosomes,” “cell-derived microparticles,” and “osteoporosis”) and relevant free-text terms were used. Detailed search strategies for each database are provided in the Supplementary Materials (Table S1). All retrieved records were imported into EndNote for duplicate removal. Study selection was conducted in two stages. First, titles and abstracts were screened to identify potentially eligible studies. Subsequently, full-text articles were assessed for final inclusion according to predefined eligibility criteria.

### 2.3. Study Selection Criteria

Studies were included if they met all of the following criteria: (1) The study involved in vivo animal experiments evaluating the therapeutic effects of stem cell-derived EVs on osteoporosis; (2) The study was designed as a controlled interventional study; (3) The study reported at least one of the following outcome measures: BMD, bone volume (BV) or bone volume fraction (BV/TV), trabecular number (Tb. N), trabecular thickness (Tb. Th), trabecular separation (Tb. Sp) and cortical thickness (Ct. Th).

Studies were excluded if they met any of the following criteria: (1) the article was not published in English; (2) the publication was not a full-text controlled interventional study (e.g., conference abstract, review, case report, editorial, comment, or letter); (3) the study did not include in vivo animal experiments; (4) the intervention group did not use actual EVs (e.g., EV mimetics or synthetic vesicles); (5) the intervention group included EVs combined with independent active therapeutic agents for osteoporosis (however, engineered EVs loaded with therapeutic cargo were eligible for inclusion when the therapeutic effects of the EV-based delivery system were evaluated); (6) the study lacked relevant outcome data, such as those listed above.

### 2.4. Risk of Bias Assessment of Included Studies

The methodological quality of the included studies was assessed using the Systematic Review Centre for Laboratory Animal Experimentation (SYRCLE) risk of bias tool [20].

### 2.5. Data Extraction

Data extraction was conducted using a predefined and standardized data extraction form. Extracted information included EV characteristics (e.g., EV subtypes, parental cell sources, and EV isolation and characterization methods), EV engineering strategies (e.g., endogenous and exogenous engineering approaches), animal model characteristics (e.g., species and disease models), and intervention details (e.g., EV dosage, delivery route, and administration frequency). Outcome data encompassed major findings from both in vitro and in vivo experiments, as well as reported molecular mechanisms. Where necessary, corresponding authors were contacted to obtain missing or unclear data.

### 2.6. Data Analysis

Due to substantial heterogeneity in experimental designs across the included studies, including variations in EV sources and subtypes, engineered versus non-engineered EVs, animal models, EV dosages, and administration routes, a quantitative meta-analysis could not be appropriately conducted. Therefore, a qualitative synthesis of the findings was performed.

## 3. Results

### 3.1. Systematic Review

**Figure 1:**
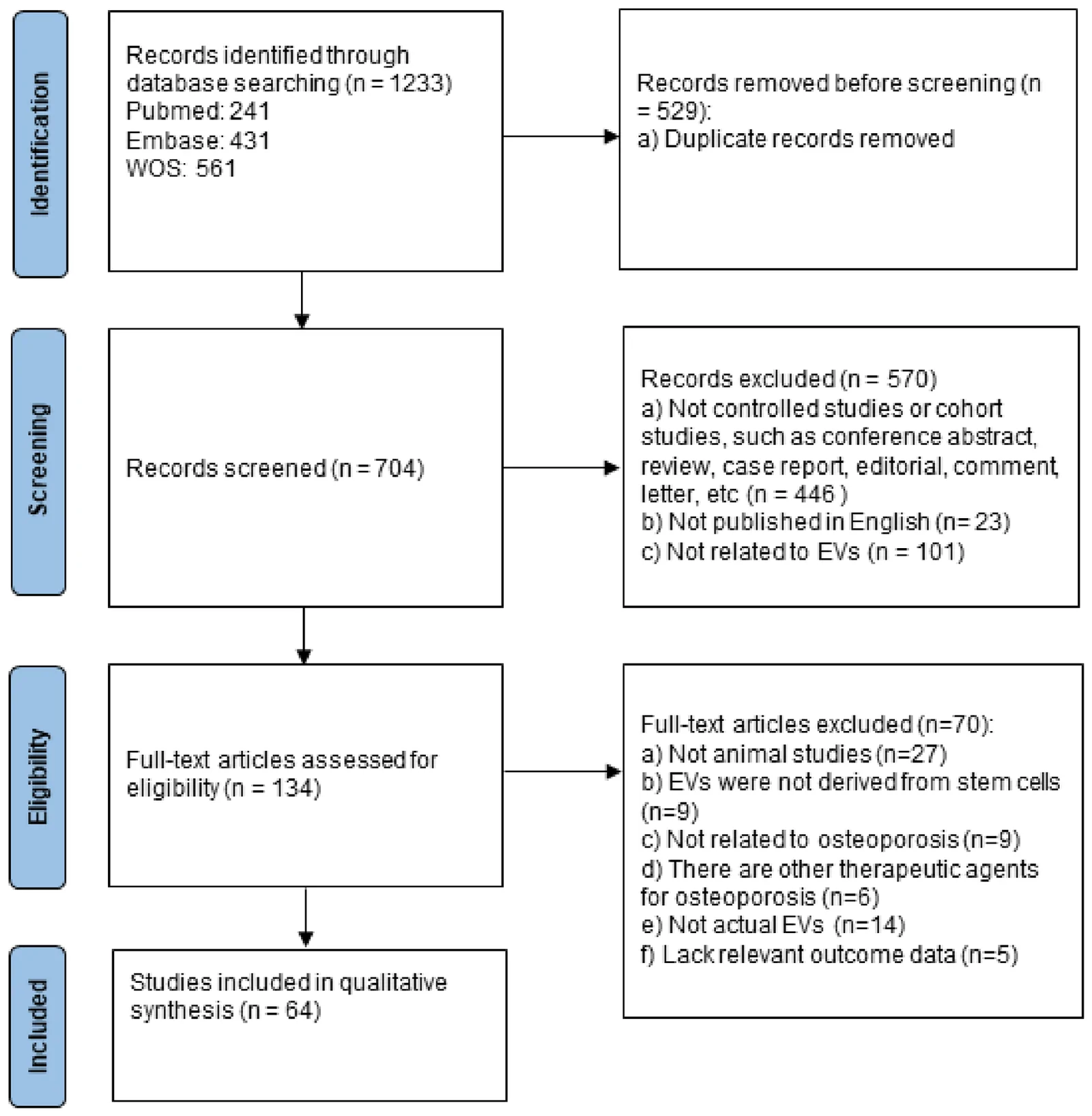
PRISMA flow diagram of literature search and selection of studies for systematic review.

**Figure 2:**
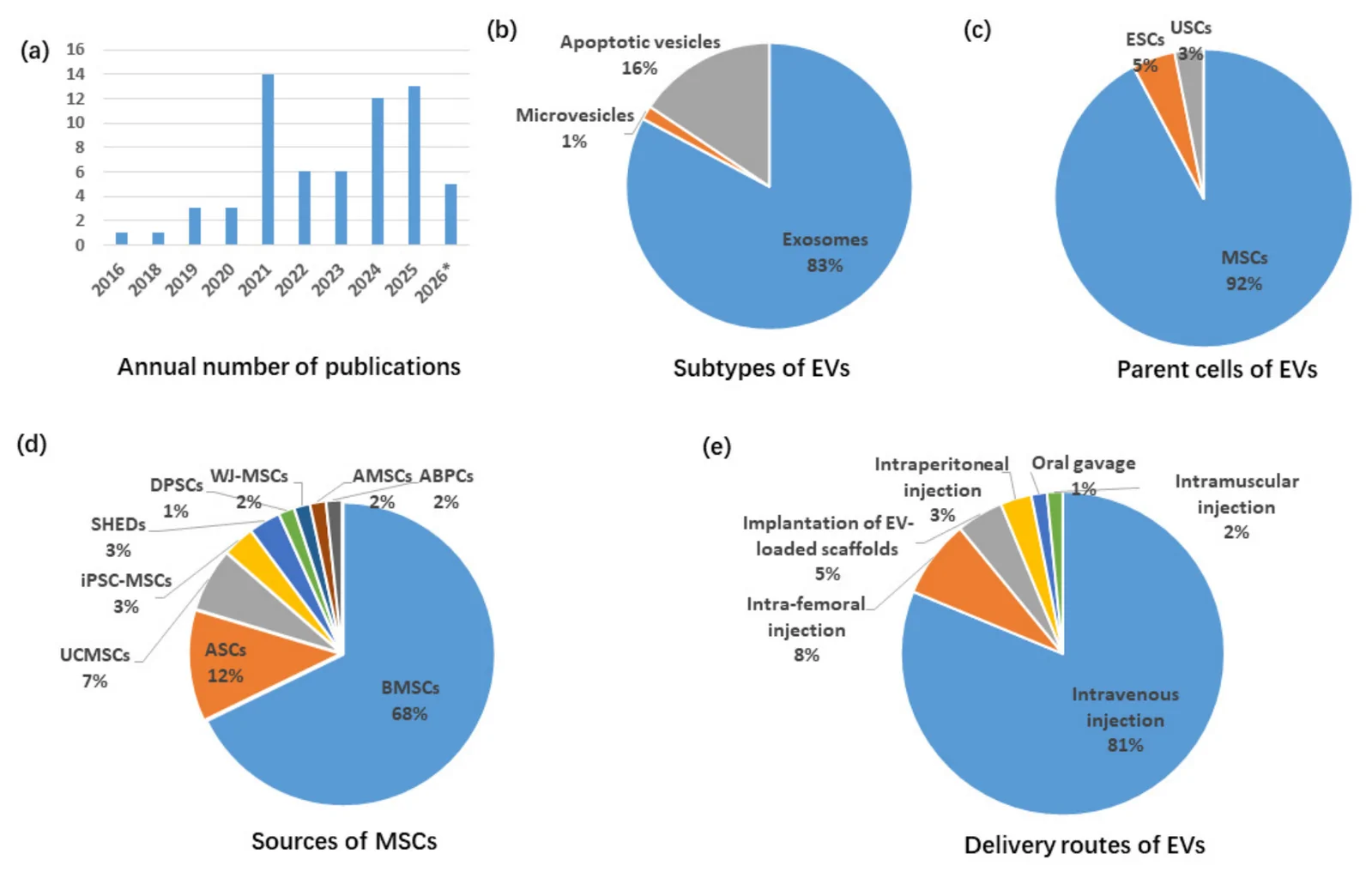
Summary of studies on stem cell-derived EVs for osteoporosis treatment. (**a**) Annual publication trend of included studies. (**b**) Subtypes of EVs used in the included studies. (**c**) Classification of EV parental cell types. (**d**) Sources of MSCs for EV isolation. (**e**) Routes of EV administration employed in the included studies. * Data for 2026 were collected up to 5 March 2026.

As illustrated in Figure 1, a total of 1233 records were identified through database searches, including 241 from PubMed, 431 from Embase, and 561 from Web of Science. After removal of duplicates, 704 records remained for title and abstract screening. Following title and abstract screening, 570 records were excluded, and 134 articles underwent full-text assessment. During full-text review, 70 articles were excluded based on the predefined eligibility criteria. Ultimately, 64 studies met the eligibility criteria and were included in this systematic review [21,22,23,24,25,26,27,28,29,30,31,32,33,34,35,36,37,38,39,40,41,42,43,44,45,46,47,48,49,50,51,52,53,54,55,56,57,58,59,60,61,62,63,64,65,66,67,68,69,70,71,72,73,74,75,76,77,78,79,80,81,82,83,84]. The included studies were published from 2016 onwards, showing a rapidly increasing trend. The annual publication trend is presented in Figure 2a.

The detailed characteristics of the included studies are summarized in the Supplementary Materials (Tables S2 and S3). Table S2 provides comprehensive information on EV subtypes and sources, isolation and characterization methods, engineering strategies, and major in vitro findings. Table S3 summarizes the animal models used, EV administration routes and dosages, principal in vivo outcomes, and relevant molecular mechanisms where applicable.

### 3.2. Risk of Bias Assessment

The results of the risk of bias assessment for each included study are presented in the Supplementary Materials (Table S4). For sequence generation, most studies reported that experimental animals were randomly allocated but did not describe the specific methods used to generate the random sequence; therefore, this domain was rated as having an unclear risk of bias. For baseline characteristics, although the majority of studies reported basic animal characteristics, such as strain, sex, age, and body weight, they did not provide information on baseline comparability after group allocation. Consequently, this domain was also rated as having an unclear risk of bias. Only one study explicitly reported no significant differences in baseline BMD or related parameters between groups before EV administration and was therefore rated as having a low risk of bias in this domain [79]. For performance bias, only one study mentioned that the treatment administrators were blinded to group allocation during the experiment, and this domain was accordingly rated as having a low risk of bias [79]; the remaining studies were rated as having an unclear risk of bias due to insufficient information. For detection bias, six studies clearly stated that outcome assessors were blinded to group allocation and were therefore rated as having a low risk of bias, whereas the remaining studies were rated as having an unclear risk of bias [54,57,72,79,81,83]. None of the studies reported random housing or random outcome assessment; thus, both domains were judged as having an unclear risk of bias. For attrition bias, only three studies provided sufficient information to assess potential animal attrition and outcome completeness, such as explicitly reporting animal deaths and their causes or confirming that all animals completed the experiment without exclusions; these studies were therefore rated as having a low risk of bias [54,60,79]. The remaining studies were classified as having an unclear risk of bias because outcome completeness and potential animal attrition could not be adequately assessed. For selective reporting bias, this domain was assessed based on whether the outcomes described in the Section 2 were consistently reported in the Section 3. Based on this criterion, 63 of the 64 included studies were rated as having a low risk of bias in this domain, while one study was judged as having an unclear risk due to insufficient information for complete evaluation [39].

### 3.3. Subtypes and Sources of EVs

Traditionally, EV nomenclature and classification have largely relied on biogenesis-based criteria, categorizing EVs into three major categories: exosomes, microvesicles, and apoptotic bodies. However, according to the latest ISEV consensus, the use of biogenesis-based terminology is discouraged unless specific EV subpopulations have been rigorously isolated and characterized. Instead, the generic term “EVs” is recommended [14]. Nevertheless, exosome-related and other subtype-specific terms remain widely used in the existing literature. Among the 64 studies included in this review, 33 used the term “exosomes”, 21 used “EVs”, and the remaining 10 employed “apoptotic vesicles” for the treatment of osteoporosis. Therefore, for the purpose of data synthesis and comparison in this review, studies using the generic term “EVs” were further classified according to the EV subtype characteristics reported in the original studies. Accordingly, the EV subtype distribution presented in Figure 2b follows the conventional biogenesis-based classification scheme. In recent years, the concept of apoptotic vesicles has expanded beyond the traditional view of apoptotic bodies, recognizing that apoptotic cells release a heterogeneous spectrum of EVs with diverse sizes and biological properties. Early studies primarily focused on apoptotic bodies, which are relatively large vesicles typically ranging from 1 to 5 μm in diameter. However, accumulating evidence has demonstrated that apoptotic vesicles constitute a heterogeneous group of vesicles with diverse sizes, including apoptotic bodies (approximately 1–5 μm), apoptotic microvesicles (approximately 0.1–1 μm), and apoptotic exosomes (<150 nm in diameter) [27]. These vesicles contain diverse bioactive cargo derived from their parent cells, including nucleic acids, proteins, and lipids, and have been increasingly recognized for their therapeutic potential in various diseases [85]. Notably, all ten studies included in our review reported beneficial effects of apoptotic vesicles in osteoporosis models.

This systematic review investigates the therapeutic efficacy of stem cell-derived EVs in the treatment of osteoporosis. Among the 64 included studies, the species origins of EV-producing cells included humans (n = 28), rats (n = 12), mice (n = 22), and deer (n = 1), while one study did not specify the species of origin. As summarized in Figure 2c, the majority of studies (n = 59) employed MSCs as the EV source, followed by embryonic stem cells (ESCs) in three studies and urine-derived stem cells (USCs) in two studies. Among MSC-based studies, bone marrow-derived MSCs (BMSCs) were the most frequently used source (n = 40), followed by adipose-derived MSCs (ASCs; n = 7) and umbilical cord-derived MSCs (UCMSCs; n = 4). Other MSC sources included induced pluripotent stem cell-derived MSCs (iPSC-MSCs; n = 2) and stem cells from human exfoliated deciduous teeth (SHEDs; n = 2), while dental pulp stem cells (DPSCs), Wharton’s jelly-derived MSCs (WJ-MSCs), amniotic membrane-derived MSCs (AMSCs), and antler blastema progenitor cells (ABPCs) were each reported in a single study. A detailed summary of MSC sources is presented in Figure 2d.

Although MSC-derived EVs represent the predominant EV source investigated for osteoporosis therapy, accumulating evidence suggests that EV biological activity is closely associated with the origin and biological state of parental cells. Among different stem cell sources, Qu et al. compared ESC-derived apoptotic vesicles with UCMSC-derived apoptotic vesicles and found that ESC-derived apoptotic vesicles exhibited superior therapeutic effects in osteoporosis models, suggesting that differences in parental cell sources may contribute to variations in EV therapeutic efficacy [23]. In addition to cell origin, variations in parental cell state and preconditioning conditions may also influence EV functional properties. For example, EVs derived from osteogenically induced ASCs or UCMSCs exhibited enhanced osteogenic potential compared with EVs from untreated cells [41,67], while EVs derived from mechanically stimulated BMSCs also showed enhanced therapeutic effects in a disuse osteoporosis model [40]. Furthermore, donor age may represent another factor affecting EV function, as EVs derived from BMSCs isolated from young mice exhibited stronger osteogenic activity and therapeutic effects than those derived from aged mice [71]. Collectively, these findings indicate that the therapeutic properties of EVs are closely associated with the characteristics of their parental cells, including cell origin and biological state.

### 3.4. Isolation and Characterization of EVs

Regarding EV isolation methods, conventional differential ultracentrifugation was the most commonly used isolation method (n = 40). Other methods included differential centrifugation for apoptotic vesicles (n = 10), commercial isolation kits (n = 10), ultrafiltration (n = 2), and density gradient ultracentrifugation (n = 1). Notably, one study compared EVs isolated by differential ultracentrifugation with those obtained using a commercial kit in an OVX mouse model of osteoporosis and reported no statistically significant difference in therapeutic effects between the two isolation methods [65]. Although the use of EV-depleted serum was frequently reported among the included studies, explicit evaluation of non-conditioned medium controls was rarely reported. Overall, cell culture conditions and EV isolation procedures were generally well described, although the reported parameters did not consistently cover the full range of methodological details recommended by MISEV2023 [14].

Regarding EV characterization, most included studies employed two or more complementary techniques to characterize the morphology, particle size distribution, particle concentration, and molecular markers of isolated EVs. Commonly used methods included scanning or transmission electron microscopy (SEM/TEM), nanoparticle tracking analysis (NTA), dynamic light scattering (DLS), Western blotting, and flow cytometry. Despite variations across studies, EVs were generally reported to display characteristic lipid bilayer-enclosed vesicular structures under electron microscopy, with diameters typically ranging from 30 to 200 nm, consistent with the size range commonly reported for small EVs, and to express canonical EV-associated markers such as CD9, CD63, CD81, and TSG101. However, assessment of negative or contaminant markers, such as lipoprotein-associated contaminants and cellular organelle-associated proteins, was rarely reported, suggesting that the purity of EV preparations was not consistently evaluated. Notably, studies focusing on apoptotic vesicles described a broader size distribution (approximately 50–5000 nm) and identified apoptosis-associated markers, including annexin V, cleaved caspase-3, cleaved PARP, and calreticulin.

### 3.5. Engineering of EVs

To enhance the therapeutic performance of EVs, various engineering strategies have been developed. Current EV engineering approaches can be broadly classified into two categories: endogenous engineering and exogenous engineering. Endogenous engineering refers to the modification of EV-producing cells prior to EV isolation, whereas exogenous engineering involves direct engineering of isolated EVs [86]. Among the 64 studies included in this systematic review, 35 studies utilized naturally secreted EVs, whereas 29 studies employed engineered EVs, reflecting the increasing interest in improving EV bioactivity, targeting specificity, and therapeutic performance.

Exogenous engineering strategies have primarily been developed to enhance EV targeting efficiency or improve their therapeutic properties. Among the included studies, six employed exogenous engineering approaches, with five focusing on improving bone-targeting capability and one aiming to enhance EV functional activity. To improve bone targeting, several studies have functionalized the EV surface using different targeting moieties. For example, bone-targeting peptides were incorporated onto EV membranes through hydrophobic interactions [21], aptamers were used to enhance recognition of BMSCs [28,31], and alendronate was conjugated onto EV surfaces via click chemistry to increase bone affinity [32]. These approaches generally enhanced EV accumulation at bone sites and were associated with improved therapeutic outcomes in osteoporosis models. Beyond chemical modification, magnetic nanoparticle-based approaches have been explored for externally controlled EV targeting. Xu et al. developed CD63 antibody-functionalized gelatin magnetic nanoparticles (GMNPs), which enabled EV binding and magnetically guided accumulation at the femoral region, thereby enhancing bone targeting and therapeutic efficacy in a diabetic osteoporosis model [36]. Additionally, one study enriched Thy1-positive EVs using magnetic-activated sorting, followed by further functionalization via protein corona technology to enhance Thy1 presentation on the EV surface, thereby improving their pro-osteogenic and anti-adipogenic effects [71].

Endogenous engineering strategies are performed at the parental cell level and include preconditioning and genetic engineering approaches before EV isolation. In this review, 19 studies adopted endogenous strategies. Among these, preconditioning approaches were used in 10 studies, involving osteogenic induction, hypoxia, mechanical stimulation, or treatment with pharmacological agents or nanomaterials to parental cells before EV isolation. Osteogenic induction of ASCs and UCMSCs generated EVs with enhanced pro-osteogenic activity [41,67], whereas hypoxic preconditioning resulted in EVs enriched with growth factors and neurotrophic factors [82]. In five studies, parental cells were treated with pharmaceutical agents prior to EV isolation to enhance the bioactivity of the secreted EVs [46,62,63,75,77]. Additional preconditioning approaches, including cyclic mechanical stimulation [40] and nanomaterial-based treatment [84], were also reported to enhance the biological activity of EVs. In addition to these preconditioning strategies, nine studies employed genetic engineering approaches in parental cells using lentiviral, adenoviral, or liposomal delivery systems to induce overexpression or inhibition of specific genes or miRNAs, thereby improving the therapeutic potency of the derived EVs [34,42,45,52,64,66,76,81,83].

Beyond separately applied endogenous or exogenous engineering strategies, four studies in this review employed integrated engineering approaches that combined both strategies to enhance the therapeutic performance of EVs. These combined approaches enhanced both therapeutic potency and bone-targeting capability of engineered EVs in osteoporosis models [33,56,70,74]. For instance, Gui et al. transduced SHEDs with an adenoviral vector encoding RNF146 to enhance the pro-osteogenic capacity of the derived EVs. Following EV isolation, a bone-targeting peptide was further conjugated onto the EV membrane via hydrophobic insertion, improving their in vivo bone-targeting efficiency [33].

### 3.6. Animal Model

A total of 64 included studies employed nine types of osteoporosis animal models. The most frequently used model was the OVX-induced postmenopausal osteoporosis model (n = 37). Other models included aging-related osteoporosis (n = 9), diabetic osteoporosis (n = 5), glucocorticoid-induced osteoporosis (n = 2), hindlimb unloading (HU)-induced disuse osteoporosis (n = 2), radiation-induced osteoporosis (n = 1), ankylosing spondylitis-associated osteoporosis (n = 1), cystathionine β-synthase (CBS) deficiency-induced metabolic osteoporosis (n = 1), and spaceflight-induced osteoporosis (SFOP; n = 1). In addition, three studies employed both OVX and aging-related osteoporosis models [25,26,83], while two studies used a combination of OVX, aging, and disuse osteoporosis models [54,59]. Most included studies were conducted in small animals (mice and rats), with only one study utilizing a large animal model (rhesus macaque) [79].

### 3.7. Delivery of EVs

Considerable heterogeneity was observed across the included studies in terms of EV dosage, delivery routes, and administration frequency, thereby limiting the feasibility of direct cross-study comparisons. EV concentration was generally quantified either by total protein content or by particle number using techniques such as NTA, DLS, or flow cytometry. Among studies using protein content to define EV dosage, 100 μg per animal was the most commonly applied dose, although substantial variability was noted. Similarly, studies reporting EV dosage based on particle number exhibited a wide range from 4 × 10^6^ to 1 × 10^12^ particles per animal. Regarding delivery routes, intravenous injection was the predominant method (n = 52). Alternative strategies included intra-femoral injection (n = 5), local implantation of EV-loaded scaffolds at bone defect sites (n = 3), intraperitoneal injection (n = 2), oral gavage (n = 1), and intramuscular injection (n = 1). A summary of EV delivery routes is presented in Figure 2e.

### 3.8. In Vitro and In Vivo Results

**Figure 3:**
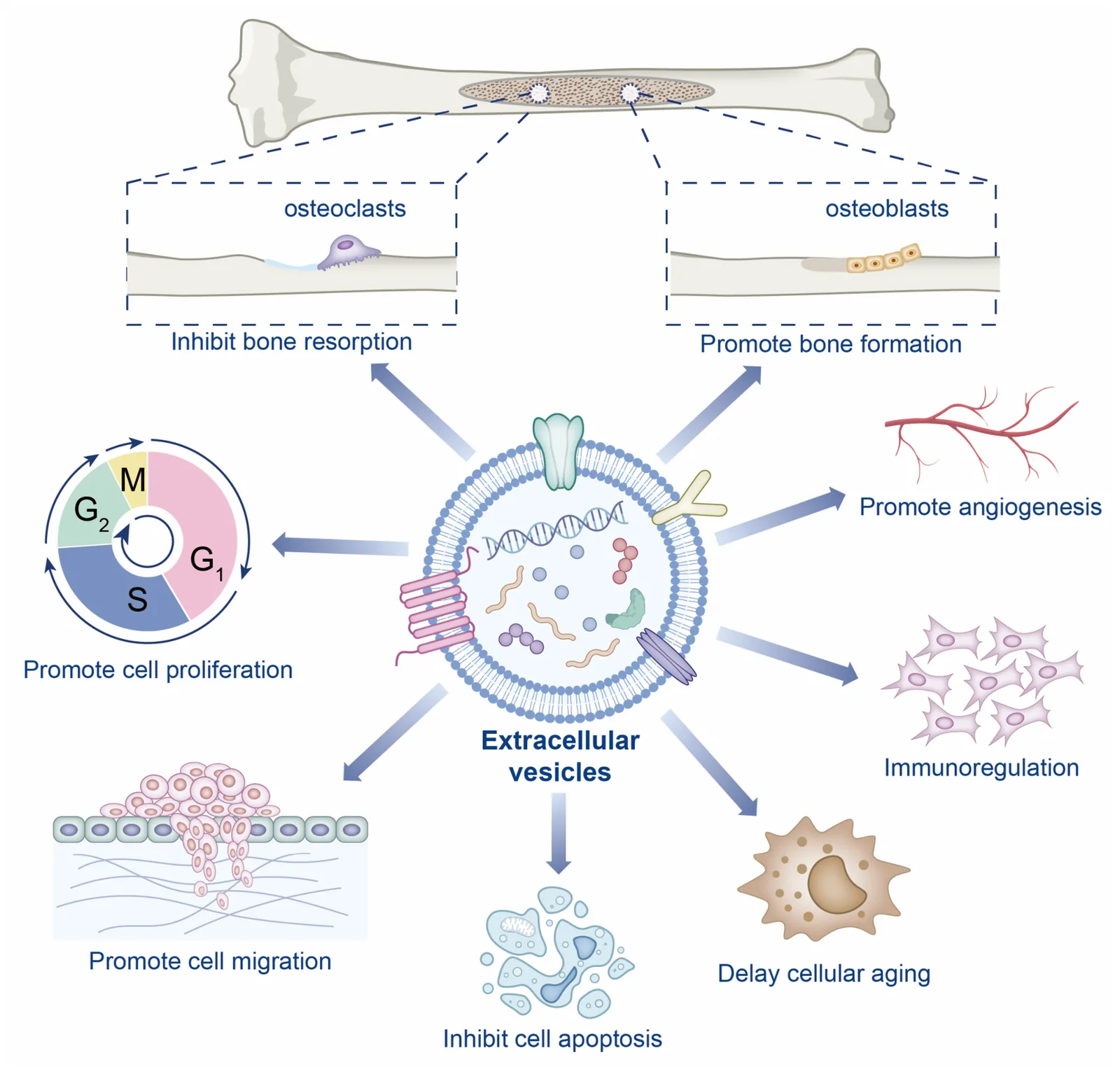
Pleiotropic effects of stem cell-derived EVs in the treatment of osteoporosis.

Among the 64 studies included in this review, 63 reported beneficial effects of native or engineered stem cell-derived EVs in osteoporosis models [21,22,23,24,25,26,27,28,29,30,31,32,33,34,35,36,37,38,39,40,41,42,43,44,45,46,47,48,49,50,51,52,53,54,55,56,57,58,59,60,61,62,63,64,65,66,67,68,69,70,71,72,73,74,75,76,77,78,79,81,82,83,84]. Evidence from in vitro and in vivo studies indicates that these EVs exert pleiotropic biological effects, including the enhancement of osteogenic differentiation; suppression of osteoclastogenesis; promotion of cell survival, proliferation, and migration; inhibition of apoptosis; attenuation of cellular senescence; stimulation of angiogenesis; and modulation of inflammatory responses, as illustrated in Figure 3. Notably, only one study reported contrary findings. In this study, EVs were derived from BMSCs of patients with osteoporosis. These EVs were shown to inhibit angiogenesis and suppress the osteogenic differentiation of hFOB1.19 cells in vitro, and to exacerbate bone loss in an OVX-induced osteoporosis model in vivo [80].

#### 3.8.1. Enhancement of Osteogenic Differentiation

A central pathological feature of osteoporosis is the disruption of the dynamic balance between bone formation and bone resorption. A large number of studies included in this review demonstrated that stem cell-derived EVs promote osteogenic differentiation of various cell types in vitro, including MSCs [22,23,25,26,35,38,39,47,49,51,54,57,58,59,61,65,69,78,79], pre-osteoblasts [50,60,72], and osteoblasts [30,37]. This was evidenced by increased alkaline phosphatase (ALP) activity, enhanced mineralized matrix formation, and upregulated expression of osteogenesis-related genes and proteins, such as RUNX2, osteocalcin (OCN), osteopontin (OPN), and bone morphogenetic protein 2 (BMP2). In addition, stem cell-derived EVs modulated the osteogenic-adipogenic differentiation balance of MSCs by promoting osteogenesis while suppressing adipogenesis [23,59,69,71].

In animal models of osteoporosis, the therapeutic effects of stem cell-derived EVs were evaluated using multiple complementary assessments, including radiological imaging, histological analyses, and biomechanical testing. At the morphological level, micro-computed tomography (micro-CT) and X-ray imaging were commonly used to quantitatively assess skeletal structural changes following EV treatment. Across the reviewed studies, EV treatment was generally associated with improvements in bone mass and trabecular microarchitecture, although the parameters evaluated and the observed improvements varied among studies. Frequently reported micro-CT outcomes included increases in BV/TV, BMD, Tb. N, and Tb. Th, as well as reductions in Tb. Sp, although not all of these parameters were assessed in every study [21,22,23,24,25,26,27,28,29,30,31,32,33,34,35,36,37,38,39,40,41,42,43,44,45,46,47,48,49,50,51,52,53,54,55,56,57,58,59,60,61,62,63,64,65,66,67,68,69,70,71,72,73,74,75,76,77,78,79,81,82,83,84]. Detailed outcome measurements reported by individual studies are summarized in Table S3. At the histological level, bone tissue changes following EV treatment were evaluated using methods such as hematoxylin and eosin (H&E) staining, Masson’s trichrome staining, immunohistochemistry, and fluorochrome labeling. EV treatment generally ameliorated osteoporotic phenotypes, with commonly reported improvements including increased bone area and improved trabecular structure and organization; however, the reported histological improvements varied across studies [22,24,25,26,27,28,29,30,31,34,35,36,37,38,39,40,41,44,47,49,50,51,52,54,55,56,57,58,59,60,62,63,64,65,66,67,69,70,71,72,74,75,76,77,78,79,81,83,84]. A considerable number of studies also reported increased osteoblast and osteocyte numbers and/or enhanced osteogenic activity following EV treatment [22,26,28,31,39,41,47,49,52,54,55,56,59,62,63,65,66,69,70,74,75,77,78,81,84], while several studies demonstrated reduced bone marrow adiposity [27,29,31,50,59,65,66,72,76]. Additionally, fluorochrome labeling demonstrated increased mineral apposition rate in EV-treated groups, reflecting enhanced bone formation activity [21,26,35,47,49,50,64,79]. At the functional level, biomechanical testing further confirmed the beneficial effects of EVs on bone mechanical properties, as evidenced by improved mechanical strength and enhanced fracture resistance [38,53,54,59,64,77,79,82]. Collectively, these findings indicate that stem cell-derived EVs ameliorate osteoporotic phenotypes and improve bone microarchitecture and mechanical properties.

#### 3.8.2. Suppression of Osteoclastogenesis

In addition to promoting osteogenesis, stem cell-derived EVs inhibit osteoclastogenesis both in vitro and in vivo. The included studies demonstrated that these EVs suppressed the differentiation of RAW264.7 cells and primary bone marrow-derived macrophages into osteoclasts in vitro, accompanied by downregulation of key osteoclast-related markers, including tartrate-resistant acid phosphatase (TRAP), nuclear factor of activated T cells 1 (NFATc1), matrix metalloproteinase 9 (MMP-9), and cathepsin K (CTSK) [21,26,36,40,43,45,48,50,54,59,60,66,84]. In osteoporotic animal models, TRAP staining revealed a reduced number of osteoclasts in EV-treated bone tissues [21,23,26,35,36,40,41,42,49,50,51,54,55,56,59,65,66,84]. Immunohistochemical analyses further demonstrated decreased expression of osteoclast activity markers, such as TRAP, MMP-9, and CTSK [75,76]. In addition, ELISA-based quantification of serum biomarkers indicated that EV treatment attenuated bone resorptive activity in vivo, as reflected by reduced levels of bone resorption markers (e.g., C-terminal telopeptide of type I collagen, CTX-1) and osteoclast-associated markers (e.g., TRAP5b) [21,35,36,51,54,56,60,66].

#### 3.8.3. Regulation of Cell Survival, Proliferation, Migration, Apoptosis, and Cellular Senescence

Bone homeostasis is severely disrupted in the context of osteoporosis. Under pathological conditions such as aging, estrogen deficiency, and glucocorticoid excess, the bone marrow microenvironment undergoes profound alterations characterized by elevated oxidative stress, immune dysregulation, increased cellular senescence, and impaired angiogenesis [4]. These alterations collectively impair osteogenic differentiation of MSCs and other osteogenic lineage cells, while promoting apoptosis and suppressing proliferation and migration.

Against this pathological background, impaired cell proliferation constitutes an important constraint on bone tissue regeneration. In osteoporosis, the reduced proliferative capacity of MSCs and osteogenic lineage cells directly contributes to insufficient bone formation [87]. Evidence from the included studies demonstrated that stem cell-derived EVs promoted the proliferation of MSCs and osteoblastic cells, including both primary cells and established cell lines (e.g., hFOB1.19 and MG-63). This pro-proliferative effect was supported by increased expression of proliferating cell nuclear antigen (PCNA), a reduced proportion of cells in the G0/G1 phase, an increased proportion of cells in the S and G2/M phases, and elevated percentages of BrdU-positive cells [23,29,32,34,35,37,38,39,46,47,49,52,55,56,57,58,62,67,68,70,75,77,78,79,82].

In addition to regulating cell proliferation, stem cell-derived EVs also promote the migration and recruitment of MSCs and osteoprogenitor cells to sites of bone remodeling, a process essential for effective bone formation. Evidence from the included studies indicated that EVs enhanced the migratory capacity of MSCs in vitro, as demonstrated by Transwell assays [48,49,64]. In vivo, EVs were shown to promote the chemotactic homing of host MSCs to bone tissues, as revealed by immunofluorescence analyses, thereby facilitating their recruitment to bone remodeling sites, which may contribute to bone regeneration [26].

Beyond their effects on proliferation and migration, stem cell-derived EVs also play a critical role in maintaining bone homeostasis by regulating cell survival. In osteoporosis, excessive apoptosis of MSCs and osteogenic lineage cells leads to depletion of the osteogenic cell population, thereby further impairing bone formation [4]. Stem cell-derived EVs were shown to attenuate apoptosis in MSCs, primary osteoblasts, and osteoblastic cell lines (e.g., MC3T3-E1 cells) in vitro, as evidenced by decreased proportions of both early apoptotic (Annexin V^+^/PI^−^) and late apoptotic/dead (Annexin V^+^/PI^+^) cell populations. This effect was accompanied by downregulation of pro-apoptotic markers, including Bax and cleaved caspase-3, and upregulation of the anti-apoptotic factor Bcl-2 at both the gene and protein levels. Furthermore, TUNEL staining of bone tissue demonstrated that EV treatment significantly reduced the number of apoptotic cells in vivo, supporting their pro-survival role within the bone microenvironment [34,44,53].

Cellular senescence is also recognized as a critical contributor to the progression of osteoporosis [88]. Evidence from the included studies indicated that stem cell-derived EVs attenuated MSC senescence in vitro, as evidenced by enhanced colony-forming unit (CFU) capacity, reduced proportions of SA-β-gal-positive cells, and downregulation of senescence-associated markers, including p53, p21, and p16. In aged osteoporotic animal models, these anti-senescent effects were further supported by decreased expression of senescence-associated proteins (e.g., p21 and p16) and reduced SA-β-gal staining in bone tissues, along with lower circulating levels of senescence-associated secretory phenotype (SASP) factors as measured by ELISA, collectively indicating attenuation of cellular senescence in vivo [23,24,27,29,53,57,58,74,79].

#### 3.8.4. Promotion of Angiogenesis

Angiogenesis is essential for bone regeneration, as adequate vascular perfusion ensures sufficient oxygen and nutrient supply [89]. Stem cell-derived EVs, particularly those derived from MSCs, exhibit potent pro-angiogenic effects [16,90]. The included studies demonstrated that these EVs promoted endothelial cell proliferation, migration, and tube formation in vitro. In osteoporotic animal models, EV treatment further enhanced bone vascularization, as evidenced by increased expression of vascular markers detected by immunofluorescence and immunohistochemistry, as well as enhanced vascular network formation assessed by Microfil perfusion combined with micro-CT imaging [21,38,47,84].

#### 3.8.5. Regulation of Inflammatory Responses

In osteoporosis, the bone microenvironment is characterized by a chronic inflammatory state, accompanied by increased inflammatory cell infiltration and elevated levels of pro-inflammatory cytokines, which disrupt the balance between bone formation and resorption and accelerate disease progression [4]. Evidence from the included studies indicated that stem cell-derived EVs exerted immunomodulatory effects by promoting macrophage polarization toward the anti-inflammatory M2 phenotype [30,84], suppressing Th17 cell differentiation, enhancing regulatory T cell (Treg) differentiation [72,76], and inhibiting inflammatory cytokine production as well as NLRP3 inflammasome activation [43,45]. In osteoporotic animal models, EV treatment further reduced inflammatory cytokine levels in serum, bone marrow and bone tissue, supporting the anti-inflammatory effects of EV treatment in vivo [45,68,79,84].

### 3.9. Potential Molecular Mechanisms

As described above, stem cell-derived EVs exert multifaceted therapeutic effects in the treatment of osteoporosis. These biological activities are largely attributed to their function as natural mediators of intercellular communication, enabling the transfer of diverse bioactive cargo, including nucleic acids, proteins, and lipids, to recipient cells, thereby modulating cellular phenotype and function [15]. To investigate the molecular mechanisms underlying the therapeutic effects of EVs in osteoporosis, many studies included in this review employed omics-based approaches, including proteomic analyses, transcriptomic profiling (mRNA, lncRNA, circRNA, and miRNA), and public database mining combined with bioinformatic analyses, to identify candidate EV cargo and associated regulatory mechanisms. In a subset of studies, these candidate molecules were further validated through functional assays. The key experimentally validated EV cargo and their therapeutic roles are summarized in Table 1.

Among the studies included in this review, 13 employed proteomic approaches to characterize protein profiles in EVs, EV-producing parental cells, or EV-treated recipient cells, followed by bioinformatic analysis to identify candidate proteins associated with EV-mediated therapeutic effects [23,24,49,53,54,55,57,58,59,71,75,79,82]. Of these, 9 studies further validated candidate proteins through gain- and loss-of-function experiments or pharmacological interventions [23,49,53,54,55,58,59,71,75]. The functionally validated EV-associated proteins were mainly involved in regulating osteogenic differentiation, osteoclastogenesis, and cellular senescence. For example, Ras, CTHRC1, Sema3A, and Thy1 were reported to promote osteogenesis [49,54,71,75], whereas osteoprotegerin (OPG) was associated with inhibition of osteoclastogenesis [54]. Notably, CLEC11A exhibited multifunctional regulatory effects by modulating the osteogenic-adipogenic balance of MSCs through promoting osteogenesis and suppressing adipogenesis, while also exerting inhibitory effects on osteoclastogenesis [59]. In addition, TCOF1, PLAU, and TIMP1 were implicated in anti-senescent effects [23,53]. Beyond these functions, STX5 was reported to contribute to Golgi apparatus repair, suggesting additional roles of EV-associated proteins in maintaining cellular homeostasis [55].

At the transcriptomic level, 10 studies compared RNA expression profiles between osteoporotic patients or animal models and healthy controls, or between EV-treated and untreated recipient cells or tissues, using RNA sequencing, PCR arrays, or public database mining [22,23,35,36,56,57,65,74,79,84]. These studies identified differentially expressed genes and signaling pathways potentially associated with EV-mediated regulation. Among them, three studies further validated candidate genes through gain- and loss-of-function experiments. The functionally validated genes were mainly associated with osteogenic differentiation, osteoclast regulation, and cellular senescence. For example, NR4A3 promoted osteoblast differentiation while inhibiting osteoclastogenesis [56], Prkar2a enhanced osteogenesis and alleviated cellular senescence [79], whereas GFAP promoted osteoclastogenesis [36]. Pathway enrichment analyses identified several canonical signaling pathways potentially involved in EV-mediated therapeutic effects, including Wnt/β-catenin, PI3K/Akt, STAT3, MAPK, and AMPK signaling pathways [22,23,35,57,65,74,84]. Importantly, several of these predicted pathways have been further validated through functional experiments. For example, MSC-derived EVs enhanced osteogenic differentiation of recipient MSCs by activating the Wnt/β-catenin signaling pathway via RNF146- and miR-328-3p-mediated suppression of Axin1 expression [35]. In addition, modulation of the PI3K/Akt pathway has been implicated in the therapeutic effects of EVs generated through different engineering strategies. Specifically, f-CA(OH) nanomaterial-preconditioned BMSC-derived EVs inhibited osteoclast differentiation through the suppression of the PI3K/Akt pathway [84], whereas cordycepin-loaded EVs regulated the PI3K/Akt/FOXO3 axis, reducing FOXO3 phosphorylation and promoting its nuclear translocation, thereby enhancing antioxidant, anti-senescent, and pro-osteogenic effects in BMSCs [74]. Transcriptomic analyses also suggested that EV treatment was associated with changes in diverse biological processes in recipient cells, including osteogenesis, stem cell proliferation, cellular senescence, osteoclast differentiation, calcium ion transport, and RNA processing [22,23,57,65].

In addition to proteomic and transcriptomic analyses of EVs, increasing attention has been directed toward miRNAs cargo within EVs. Among the included studies, 16 performed miRNA expression profiling in EVs, their parental cells, or EV-treated recipient cells using miRNA sequencing, microarray analysis, or public database mining, combined with bioinformatic approaches to identify candidate regulatory miRNAs [21,28,39,41,48,60,61,62,63,65,66,67,68,73,76,83]. Of these, 12 studies further validated the regulatory roles of these miRNAs through gain- and loss-of-function experiments [28,39,41,48,60,61,62,63,68,73,76,83], while 8 additionally confirmed downstream target genes using assays such as luciferase reporter and RNA pull-down assays [39,41,60,61,62,63,73,76]. The functionally validated EV-associated miRNAs were mainly involved in regulating osteogenesis, osteoclastogenesis, immunomodulation, and cellular senescence. For example, several miRNAs, including miR-26a, miR-186, miR-27a, miR-21-5p, miR-136-3p, miR-146a-5p, and miR-509-5p, promoted osteogenic differentiation [28,39,60,61,62,68,73], whereas miR-335-3p, miR-330-5p, and miR-4485-3p exhibited inhibitory effects on osteogenesis [41,63,83]. In addition, miR-133b-3p was reported to contribute to immunomodulation by promoting Treg cell differentiation [76]. Some EV-associated miRNAs exhibited multifunctional regulatory effects across different biological processes. For example, miR-21-5p and miR-27a additionally suppressed osteoclastogenesis [48,60], while miR-136-3p and miR-146a-5p attenuated cellular senescence [68]. Further bioinformatic analyses and experimental validation suggested that these miRNAs were involved in regulating multiple signaling pathways, including Wnt/β-catenin, PI3K/Akt, NF-κB, and Hippo pathways, thereby contributing to EV-mediated regulation of bone remodeling and related biological processes.

Long non-coding RNAs (lncRNAs) are regulatory non-coding RNAs that modulate gene expression through diverse mechanisms, including acting as competing endogenous RNAs to sequester miRNAs and relieve miRNA-mediated repression of target mRNAs. Recent studies have shown that lncRNAs are enriched and stably present in EVs and can be transferred to recipient cells to regulate cellular functions [91]. In this review, one study identified lnc-H19 as a differentially expressed EV-associated lncRNA between EVs derived from wild-type and CBS+/− BMSCs. Mechanistically, lnc-H19 delivered by EVs promoted osteogenesis and angiogenesis through the lnc-H19/miR-106a/Angpt1/Tie2-NO signaling axis [38].

Similarly, circular RNAs (circRNAs), a class of covalently closed non-coding RNAs, have been reported to act as miRNA sponges and regulate gene expression [92]. In this review, one study identified hsa_circ_0028877 as an EV-associated circRNA involved in osteogenic regulation. Functional validation demonstrated that ASC-derived EVs promoted osteogenic differentiation by inducing nuclear retention of hsa_circ_0028877 and subsequently activating the Hedgehog signaling pathway through the miR-4728-3p/PTCH1 axis [78].

Besides proteins and nucleic acids, lipids have also been investigated as potential functional components of EVs. One study performed lipidomic analysis of AMSC-derived EVs and identified phosphatidylcholine and ceramide as the predominant lipid components, suggesting that EV lipid composition may contribute to their biological properties [82].

## 4. Discussion

This systematic review provides a comprehensive overview of current preclinical research on stem cell-derived EV-based therapies for osteoporosis. Collectively, the available evidence demonstrates that EVs exert promising osteoprotective and bone-regenerative effects across various animal models of osteoporosis. However, despite these encouraging findings, substantial inter-study heterogeneity, incomplete mechanistic understanding, and considerable barriers to clinical translation remain major challenges in this field. This discussion aims to synthesize the key findings, interpret their biological and translational implications, critically address current limitations, and provide insights for future investigation.

With the exception of one study employing EVs derived from BMSCs of osteoporotic patients [80], the studies included in this review generally demonstrated that EVs derived from diverse stem cell sources, including MSCs, ESCs, and USCs, improve BMD, trabecular microarchitecture, and biomechanical properties across multiple osteoporosis models, such as ovariectomy-induced, glucocorticoid-induced, aging-related, and disuse-induced osteoporosis. Moreover, accumulating in vivo and in vitro evidence indicates that stem cell-derived EVs promote osteoporotic bone repair through multifaceted and coordinated mechanisms. In particular, these EVs enhance the osteogenic differentiation and mineralization capacity of MSCs and osteolineage cells, while simultaneously suppressing osteoclast differentiation and bone resorption. Beyond their direct regulatory effects on bone remodeling, EVs also modulate the bone marrow microenvironment to create conditions favorable for bone regeneration. Specifically, stem cell-derived EVs have been shown to improve the bone marrow microenvironment by promoting angiogenesis, modulating immune responses, enhancing cell survival, proliferation, and migration while attenuating apoptosis and cellular senescence. The biological basis underlying these multifaceted effects of EVs is primarily attributed to their role as natural mediators of intercellular communication that deliver diverse bioactive cargo, including nucleic acids, proteins, and lipids, to recipient cells, thereby inducing functional and phenotypic alterations. EVs possess a lipid bilayer membrane structure that protects their cargo from extracellular enzymes such as proteases and RNases, while also exhibiting high stability, low immunogenicity, and amenability to modification [16]. A number of studies included in this review have employed high-throughput approaches, including RNA sequencing, miRNA sequencing, and proteomic analyses, to systematically identify key bioactive molecules within EVs. Some of these candidate molecules have subsequently been validated through functional experiments. However, it is important to recognize that EVs function as integrated biological entities rather than being governed by a single key molecule. Their biological effects are mediated through synergistic interactions among diverse components, including nucleic acids (e.g., mRNAs, miRNAs, lncRNAs, and circRNAs), proteins, and lipids, which collectively form a complex regulatory network [86]. Therefore, future studies should move beyond single-molecule-centered paradigms and adopt more integrative, systems-level strategies to elucidate the coordinated mechanisms by which EVs regulate bone remodeling.

Despite the diverse biological effects of EVs described above, many studies have employed engineering strategies to further enhance their therapeutic efficacy in osteoporosis treatment. As noted, EV engineering strategies can generally be classified into two categories: endogenous engineering and exogenous engineering, both of which have been adopted in the studies included in this review. Endogenous engineering is primarily performed at the parental cell level, with preconditioning of donor cells being a commonly used strategy. For instance, osteogenic induction or pharmacological preconditioning of parental cells can significantly enhance the osteogenic potential of their secreted EVs, while being relatively simple and easy to implement. In addition, genetic engineering of parental cells to overexpress therapeutic cargo can further enhance the osteogenic potential of their EVs. However, it should be noted that the precise mechanisms underlying EV biogenesis and cargo sorting remain incompletely understood. Consequently, endogenous engineering strategies may introduce so-called “black-box effects”, potentially leading to unpredictable biological outcomes [93]. In contrast, exogenous engineering involves the direct engineering of isolated EVs and mainly includes two approaches: (1) loading exogenous therapeutic cargo, such as bioactive molecules or drugs, into EVs using techniques such as electroporation, co-incubation, sonication, extrusion, and freeze–thaw cycles; and (2) modifying the EV membrane via covalent or non-covalent interactions to display specific targeting or therapeutic moieties on the vesicle surface [94]. In the studies included in this review, EVs have been successfully functionalized with targeting moieties using strategies such as click chemistry, hydrophobic interactions, aptamer conjugation, and antigen–antibody binding, thereby substantially improving the bone-targeting efficiency of EVs following systemic administration. Compared with endogenous engineering, exogenous engineering may reduce uncertainties associated with these “black-box effects”; however, it often involves more complex procedures, which may result in EV loss as well as the introduction of by-products and impurities [93]. In addition, several studies included in this review have employed combined endogenous and exogenous engineering strategies, in which parental cells are first genetically modified to overexpress specific functional molecules, followed by EV isolation and subsequent membrane surface functionalization to introduce targeting moieties, thereby simultaneously enhancing both the therapeutic potency and targeting specificity of EVs. With the growing recognition of the therapeutic potential of EV-based therapies, increasing attention has been focused on engineering native EVs to improve their therapeutic efficacy, targeting precision, and biological performance. Future studies are expected to develop more efficient, controllable, and precise EV engineering strategies, thereby facilitating the clinical application of EV-based therapies for osteoporosis.

Previous systematic reviews and meta-analyses have provided valuable insights into the biological functions, therapeutic effects, and potential molecular mechanisms of stem cell-derived EVs in osteoporosis [9,95,96,97]. Compared with these previous systematic reviews and meta-analyses, the present systematic review places particular emphasis on several important aspects of this rapidly evolving field, including detailed characterization of parental cell sources of EVs, expanded consideration of apoptotic vesicles as an increasingly recognized EV population, comprehensive evaluation of recent advances in EV engineering strategies, and systematic analysis of EV cargo profiles and associated molecular regulatory mechanisms. A detailed comparison between the present review and previous representative systematic reviews in this field is provided in Table S5. Although stem cell-derived EVs have demonstrated considerable therapeutic potential in osteoporosis, research in this field remains at an early stage. The studies included in this review are predominantly based on small animal models (rats and mice), with only one study involving a large animal model (rhesus macaques). In addition, substantial heterogeneity exists in study design and experimental implementation, which makes direct comparisons across studies challenging. On the one hand, EVs are inherently heterogeneous, and their functional properties are largely determined by the source and physiological state of their parental cells [15,98]. On the other hand, the lack of standardized protocols for EV isolation, purification, and characterization, as well as for administration strategies (e.g., route, dosage, and frequency), further contributes to variability across studies. Regarding mechanistic investigations, some studies have identified key EV-enriched miRNAs or proteins, along with their associated downstream signaling pathways. However, studies that systematically integrate high-throughput profiling, functional validation (e.g., gain- and loss-of-function approaches), and biodistribution analyses using fluorescence tracking remain relatively limited. In terms of safety, although some studies reported animal mortality during the experimental period, these events were infrequent, and their causal relationship with EV administration remained unclear. Nevertheless, only a subset has performed systematic evaluations of potential side effects in major organs (e.g., the heart, liver, and kidney) [21,22,25,26,32,33,41,52,54,66,70,71,74,79,84]. Moreover, the quality assessment revealed that most studies were judged as having an “unclear risk” of bias in several domains, including randomization, baseline characteristics, blinding procedures and attrition bias. The predominance of “unclear risk” judgments largely reflects insufficient information regarding methodological details rather than necessarily indicating a high risk of bias. Specifically, the lack of clear descriptions of randomization procedures, outcome assessment blinding, animal attrition, and prespecified outcome protocols limited the ability to determine whether these methodological procedures were appropriately implemented. Although these methodological uncertainties limit the confidence with which the findings can be interpreted, most studies employed objective and quantitative outcome measures and reported beneficial effects following EV treatment, providing supportive evidence for their potential therapeutic value. Nevertheless, these findings should be interpreted with caution. Future studies are encouraged to adhere to standardized reporting guidelines, such as the ARRIVE 2.0 guidelines [99], to improve the transparency, reproducibility, and methodological rigor of preclinical EV research.

Furthermore, translating EV-based therapies for osteoporosis from bench to bedside still faces substantial challenges. First, establishing scalable and GMP-compatible EV manufacturing platforms remains a major challenge. Current cell culture and EV isolation methods are insufficient to meet clinical requirements in terms of yield, stability, and batch-to-batch consistency. Therefore, future studies should focus on developing standardized manufacturing workflows and scalable EV isolation technologies to enable the production of clinical-grade EV products [100]. Second, standardized characterization criteria and quality control are essential for ensuring consistent EV product quality and improving reproducibility across studies. The EV-TRACK initiative [101] and the recommendations of MISEV2023 [14] provide important guidance for enhancing reporting transparency and promoting standardized EV characterization and quality assessment. In addition, reliable potency assays are required to evaluate the biological activity of EV products, while consensus criteria for EV dose normalization need to be established to ensure accurate dose reporting and improve comparability across studies and EV batches [102]. Third, the in vivo distribution, pharmacokinetics, and bone-targeting efficiency of systemically administered EVs remain incompletely understood, highlighting the need for further investigation into EV biodistribution, tissue retention, and delivery strategies. To improve therapeutic efficacy, future studies may further optimize membrane surface modification strategies for bone targeting, as well as develop targeted and stimulus-responsive delivery systems, including magnetic field- or ultrasound-guided approaches. Finally, the long-term safety of EV-based therapies requires further investigation. Current preclinical studies with limited follow-up periods remain insufficient to comprehensively evaluate the immunogenicity, tumorigenic potential, and off-target effects of allogeneic or xenogeneic EVs. Although autologous EVs may reduce the risk of immune rejection, their preparation is labor-intensive and costly, and may be influenced by the donor’s health status, thereby potentially compromising EV bioactivity and therapeutic efficacy. Therefore, future studies are needed to systematically evaluate the efficacy and safety of EV-based therapies in large-animal models and clinical trials, and to establish standardized manufacturing procedures, quality criteria, and regulatory frameworks to facilitate clinical translation.

## 5. Conclusions

In conclusion, the studies included in this review generally demonstrate that stem cell-derived EVs represent a highly promising cell-free therapeutic strategy for osteoporosis, owing to their intrinsic biocompatibility, low immunogenicity, multifaceted biological functions, and amenability to modification. Although substantial challenges remain in translating EV-based therapies into clinical practice, continued advances in our understanding of EV biology and underlying mechanisms, together with interdisciplinary efforts to systematically address current barriers, are expected to facilitate the development of EV-based therapies as a next-generation therapeutic strategy for osteoporosis.

## Figures and Tables

**Table 1:** Key bioactive cargoes in EVs validated in the included studies.

Cargoes of EVs	Parent Cells	EVs	Therapeutic Roles and Possible Mechanisms	References
Ras	Mouse BMSCs	ApoVs	Promote osteogenic differentiation of BMSCs via Ras/Raf1/Mek/Erk signaling pathway	[29]
CTHRC1	Human USCs	Exosomes	Promote osteogenic differentiation of BMSCs	[34]
Thy1	Mouse BMSCs	ApoVs	Promote osteogenic differentiation and inhibit adipogenic differentiation of MSCs via ERK-TAZ signaling pathway	[51]
Sema3A	Mouse BMSCs pretreated with scutellaria baicalensis georgi	Exosomes	Promote osteogenic differentiation of MC3T3-E1 cells	[55]
CLEC11A	Human UCMSCs	Exosomes	Enhance the shift from adipogenic to osteogenic differentiation of BMSCs and inhibit osteoclastogenesis	[39]
OPG	Human USCs	Exosomes	Inhibit osteoclastogenesis	[34]
TCOF1	Human ESCs	ApoVs	Rejuvenate aging BMSCs via FLVCR1-mediated mitochondrial functional homeostasis	[3]
PLAU	Human USCs	Exosomes	Delay cellular aging of BMSCs	[33]
TIMP1	Human USCs	Exosomes	Delay cellular aging of BMSCs	[33]
STX5	Human SHED cell aggregate	Microvesicles	Restore Golgi homeostasis	[35]
Prkar2a	ABPCs	Exosomes	Promote osteogenic differentiation and delay cellular aging of BMSCs	[59]
miR-26a	Mouse mesenchymal stromal cells	Exosomes	Promote osteogenic differentiation of BMSCs	[8]
miR-186	Human BMSCs	Exosomes	Promote osteogenic differentiation of BMSCs via Hippo signaling pathway	[19]
miR-27a	Mouse BMSCs	Exosomes	Promote osteogenic differentiation of MC3T3-E1 cells and inhibit osteoclastogenesis via DKK2/Wnt/β-catenin signaling pathway	[40]
miR-21-5p	Human ESCs	Exosomes	Promote osteogenic differentiation of UCMSCs via YAP1/Wnt/β-catenin signaling pathway	[41]
miR-27a-5p	BMSCs pretreated with epimedium	Exosomes	Promote osteogenic differentiation of osteoblasts via targeting Atg4B	[42]
miR-136-3p	Mouse BMSCs	Exosomes	Promote osteogenic differentiation and delay cellular aging of BMSCs via PI3K-Akt signaling pathway	[48]
miR-146a-5p	Mouse BMSCs	Exosomes	Promote osteogenic differentiation and delay cellular aging of BMSCs via NF-κB signaling pathway	[48]
miR-509-5p	Human BMSCs	Exosomes	Promote osteogenic differentiation of hFOB1.19 cells via SFRP1/Wnt/β-catenin signaling pathway	[53]
miR-335-3p	Human ASCs	Exosomes	Inhibit osteogenic differentiation of BMSCs via targeting Aplnr	[21]
miR-330-5p	Rat BMSC pretreated with plastrum testudines	Exosomes	Inhibit osteogenic differentiation of pre-osteoblasts via Tnc/Wnt/β-catenin signaling pathway	[43]
miR-4485-3p	Human BMSCs	ApoVs	Inhibit osteogenic differentiation and promote adipogenic differentiation of MSCs via AKT signaling pathway	[63]
miR-133b-3p	Rat BMSCs	Exosomes	Promote CD4+CD25+Foxp3+ Treg cell differentiation via targeting TGF-β1	[56]
miR-21-5p	Human ASC	Exosomes	Inhibit osteoclastogenesis via targeting Acvr2a	[28]
lncRNA-H19	Mouse BMSCs	Exosomes	Promote angiogenesis and osteogenic differentiation of BMSCs via miR-106/Angpt1/Tie2-NO signaling pathway	[18]

## Data Availability

All data supporting the findings of this study are derived from publicly available published literature and are included within the article.

## Supplementary Materials

The following supporting information can be downloaded at: https://www.mdpi.com/article/10.3390/biomedicines14091976/s1, Table S1: Search strategy; Table S2: Summary of included studies: EV sources, isolation and characterization methods, modification strategies, and major in vitro findings [21,22,23,24,25,26,27,28,29,30,31,32,33,34,35,36,37,38,39,40,41,42,43,44,45,46,47,48,49,50,51,52,53,54,55,56,57,58,59,60,61,62,63,64,65,66,67,68,69,70,71,72,73,74,75,76,77,78,79,80,81,82,83,84]; Table S3: Summary of included studies: animal models, evaluation of EV safety and in vivo biodistribution, routes of EV administration and dosages, major in vivo findings, and underlying molecular mechanisms [21,22,23,24,25,26,27,28,29,30,31,32,33,34,35,36,37,38,39,40,41,42,43,44,45,46,47,48,49,50,51,52,53,54,55,56,57,58,59,60,61,62,63,64,65,66,67,68,69,70,71,72,73,74,75,76,77,78,79,80,81,82,83,84]; Table S4: Risk of bias assessed using SYRCLE risk of bias assessment tool [21,22,23,24,25,26,27,28,29,30,31,32,33,34,35,36,37,38,39,40,41,42,43,44,45,46,47,48,49,50,51,52,53,54,55,56,57,58,59,60,61,62,63,64,65,66,67,68,69,70,71,72,73,74,75,76,77,78,79,80,81,82,83,84]; Table S5: Comparison of the present review with previous systematic reviews and meta-analyses of stem cell-derived EVs for osteoporosis [9,95,96,97].

## Abbreviations

EVs: extracellular vesicles; PRISMA: Preferred Reporting Items for Systematic Reviews and Meta-Analyses; BMD: bone mineral density; MSCs: mesenchymal stem cells; ISEV: International Society for Extracellular Vesicles; BV: bone volume; BV/TV: bone volume fraction; Tb. N: trabecular number; Tb. Th: trabecular thickness; Tb. Sp: trabecular separation; Ct. Th: cortical thickness; SYRCLE: Systematic Review Centre for Laboratory Animal Experimentation; ESCs: embryonic stem cells; USCs: urine-derived stem cells; BMSCs: bone marrow-derived MSCs; ASCs: adipose-derived MSCs; UCMSCs: umbilical cord-derived MSCs; iPSC-MSCs: induced pluripotent stem cell-derived MSCs; SHEDs: stem cells from human exfoliated deciduous teeth; DPSCs: dental pulp stem cells; WJ-MSCs: Wharton’s jelly-derived MSCs; AMSCs: amniotic membrane-derived MSCs; ABPCs: antler blastema progenitor cells; SEM/TEM: scanning or transmission electron microscopy; NTA: nanoparticle tracking analysis; DLS: dynamic light scattering; GMNPs: gelatin magnetic nanoparticles; ALP: alkaline phosphatase; OCN: osteocalcin; OPN: osteopontin; BMP2: bone morphogenetic protein 2; micro-CT: micro-computed tomography; H&E: hematoxylin and eosin; TRAP: tartrate-resistant acid phosphatase; NFATc1: nuclear factor of activated T cells 1; MMP-9: matrix metalloproteinase 9; CTSK: cathepsin K; CTX-1: C-terminal telopeptide of type I collagen; CFU: colony-forming unit; Treg: regulatory T cell; OPG: osteoprotegerin; lncRNAs: long non-coding RNAs; circRNAs: circular RNAs.
