# Supplementary material for: Stem Cell-Derived Extracellular Vesicles for the Treatment of Osteoporosis: A Systematic Review of Preclinical Evidence
Source: Biomedicines. 2026 Sep 2;14(9):1976. doi: 10.3390/biomedicines14091976 (PMC13604492; doi:10.3390/biomedicines14091976)
Supplement: Supplementary file 1 [file biomedicines-14-01976-s001.zip › biomedicines-4431108-supplementary.pdf]

Supplementary Materials

Stem Cell-Derived Extracellular Vesicles for the Treatment of Osteoporosis: A Systematic Review of Preclinical Evidence

Yi Yang[1†], Lifen Qiao[1†], Yan Huang[2], Qin Xia[1*], Hui Xie[1*]

1. Department of Geriatrics, Tongji Hospital, Tongji Medical College of Huazhong University of Science and Technology, Wuhan, Hubei, China

2. Department of Rehabilitation Medicine, Tongji Hospital, Tongji Medical College of Huazhong University of Science and Technology, Wuhan, Hubei, China

[†]These authors contributed equally to this work.

[*]Corresponding author:

Hui Xie: E-mail: xiehuijessice@163.com.

Qin Xia: E-mail: xiaqin529@163.com

Table S1. Search strategy.

Pubmed

#	Search terms	Results
1	"Stem Cells"[Mesh]	288,541
2	(stem cells[Title/Abstract]) OR (Cells, Stem[Title/Abstract])) OR (Cell, Stem[Title/Abstract])) OR (Stem Cell[Title/Abstract])) OR (Mother Cells[Title/Abstract])) OR (Cell, Mother[Title/Abstract])) OR (Cells, Mother[Title/Abstract])) OR (Mother Cell[Title/Abstract])) OR (Progenitor Cells[Title/Abstract])) OR (Cell, Progenitor[Title/Abstract])) OR (Cells, Progenitor[Title/Abstract])) OR (Progenitor Cell[Title/Abstract])) OR (Colony-Forming Unit[Title/Abstract])) OR (Colony Forming Unit[Title/Abstract])) OR (Colony-Forming Units[Title/Abstract])) OR (Colony Forming Units[Title/Abstract])	474,245
3	"Extracellular Vesicles"[Mesh]	39,922
4	(Extracellular vesicles[Title/Abstract]) OR (Extracellular Vesicle[Title/Abstract])) OR (Vesicle, Extracellular[Title/Abstract])) OR (Vesicles, Extracellular[Title/Abstract])) OR (Exovesicles[Title/Abstract])) OR (Exovesicle[Title/Abstract])) OR (Apoptotic Bodies[Title/Abstract])) OR (Apoptotic Body[Title/Abstract])) OR (Bodies, Apoptotic[Title/Abstract])) OR (Body, Apoptotic[Title/Abstract])	39,147
5	"Exosomes"[Mesh]	20,074
6	exosomes[Title/Abstract]	34,107
7	"Cell-Derived Microparticles"[Mesh]	3,887
8	(Cell-Derived Microparticles[Title/Abstract]) OR (Cell-Derived Microparticle[Title/Abstract])) OR (Cell Derived Microparticles[Title/Abstract])) OR (Microparticle, Cell-Derived[Title/Abstract])) OR (Shedding Microvesicles[Title/Abstract])) OR (Microvesicle, Shedding[Title/Abstract])) OR (Microvesicles, Shedding[Title/Abstract])) OR (Shedding Microvesicle[Title/Abstract])) OR (Cell Membrane Microparticles[Title/Abstract])) OR (Cell Membrane Microparticle[Title/Abstract])) OR (Membrane Microparticle, Cell[Title/Abstract])) OR (Membrane Microparticles, Cell[Title/Abstract])) OR (Microparticle, Cell Membrane[Title/Abstract])) OR (Microparticles, Cell Membrane[Title/Abstract])) OR (Microparticles, Cell-Derived[Title/Abstract])) OR (Microparticles, Cell Derived[Title/Abstract])) OR (Ectosomes[Title/Abstract])) OR	2,439

	(Ectosome[Title/Abstract])) OR (Circulating Cell-Derived Microparticles[Title/Abstract])) OR (Cell-Derived Microparticle, Circulating[Title/Abstract])) OR (Cell-Derived Microparticles, Circulating[Title/Abstract])) OR (Circulating Cell-Derived Microparticle[Title/Abstract])) OR (Circulating Cell Derived Microparticles[Title/Abstract])) OR (Microparticle, Circulating Cell-Derived[Title/Abstract])) OR (Microparticles, Circulating Cell-Derived[Title/Abstract])	
9	"Osteoporosis"[Mesh]	69,115
10	(Osteoporosis[Title/Abstract]) OR (Osteoporoses[Title/Abstract])) OR (Osteoporosis, Age-Related[Title/Abstract])) OR (Osteoporosis, Age Related[Title/Abstract])) OR (Age-Related Osteoporosis[Title/Abstract])) OR (Age-Related Osteoporoses[Title/Abstract])) OR (Age Related Osteoporosis[Title/Abstract])) OR (Osteoporoses, Age-Related[Title/Abstract])) OR (Bone Loss, Age-Related[Title/Abstract])) OR (Age-Related Bone Loss[Title/Abstract])) OR (Age-Related Bone Losses[Title/Abstract])) OR (Bone Loss, Age Related[Title/Abstract])) OR (Bone Losses, Age-Related[Title/Abstract])) OR (Osteoporosis, Senile[Title/Abstract])) OR (Osteoporoses, Senile[Title/Abstract])) OR (Senile Osteoporoses[Title/Abstract])) OR (Senile Osteoporosis[Title/Abstract])) OR (Osteoporosis, Involutional[Title/Abstract])) OR (Osteoporosis, Post-Traumatic[Title/Abstract])) OR (Osteoporosis, Post Traumatic[Title/Abstract])) OR (Post-Traumatic Osteoporoses[Title/Abstract])) OR (Post-Traumatic Osteoporosis[Title/Abstract])	99,513
11	#1 OR #2	548,350
12	#3 OR #4 OR #5 OR #6 OR #7 OR #8	69,501
13	#9 OR #10	117,257
14	#11 AND #12 AND #13	241

Embase

#	Search terms	Results
1	'stem cell'/exp	563097
2	'stem cell':ti,ab,kw OR 'cell, stem':ti,ab,kw OR 'precursor cell':ti,ab,kw OR 'progenitor cell':ti,ab,kw OR 'stem cells':ti,ab,kw	636509
3	'exosome'/exp	88488
4	exosomes:ti,ab,kw OR 'extracellular vesicle':ti,ab,kw OR 'extracellular vesicles':ti,ab,kw OR exosome:ti,ab,kw	84893

5	'membrane microparticle'/exp	11758
6	'membrane microparticle':ti,ab,kw OR 'cell derived microparticle':ti,ab,kw OR 'cell derived microparticles':ti,ab,kw OR 'cell-derived microparticles':ti,ab,kw OR 'circulating microparticle':ti,ab,kw OR 'circulating microparticles':ti,ab,kw OR 'membrane microparticles':ti,ab,kw OR microparticle:ti,ab,kw OR 'membrane microvesicle':ti,ab,kw OR 'procoagulant microparticle':ti,ab,kw OR 'procoagulant microparticles':ti,ab,kw	12337
7	'osteoporosis'/exp	184101
8	osteoporosis:ti,ab,kw OR 'decalcification, pathologic':ti,ab,kw OR 'endocrine osteoporosis':ti,ab,kw OR 'osteoporotic decalcification':ti,ab,kw OR 'pathologic decalcification':ti,ab,kw	159796
9	#1 OR #2	791715
10	#3 OR #4 OR #5 OR #6	113811
11	#7 OR #8	217919
12	#9 AND #10 AND #11	431

WOS

#	**Search terms**	**Results**
1	Stem Cells (Topic) OR Cells, Stem (Topic) OR Cell, Stem (Topic) OR Stem Cell (Topic) OR Mother Cells (Topic) OR Cell, Mother (Topic) OR Cells, Mother (Topic) OR Mother Cell (Topic) OR Progenitor Cells (Topic) OR Cell, Progenitor (Topic) OR Cells, Progenitor (Topic) OR Progenitor Cell (Topic) OR Colony-Forming Unit (Topic) OR Colony Forming Unit (Topic) OR Colony-Forming Units (Topic) OR Colony Forming Units (Topic)	1279949
2	Osteoporosis (Topic) OR Osteoporoses (Topic) OR Osteoporosis, Age-Related (Topic) OR Osteoporosis, Age Related (Topic) OR Age-Related Osteoporosis (Topic) OR Age-Related Osteoporoses (Topic) OR Age Related Osteoporosis (Topic) OR Osteoporoses, Age-Related (Topic) OR Bone Loss, Age-Related (Topic) OR Age-Related Bone Loss (Topic) OR Age-Related Bone Losses (Topic) OR Bone Loss, Age Related (Topic) OR Bone Losses, Age-Related (Topic) OR Osteoporosis, Senile (Topic) OR Osteoporoses, Senile (Topic) OR Senile Osteoporoses (Topic) OR Senile Osteoporosis (Topic) OR Osteoporosis, Involutional (Topic) OR Osteoporosis, Post-Traumatic (Topic) OR Osteoporosis, Post Traumatic (Topic) OR Post-Traumatic Osteoporoses (Topic) OR Post-Traumatic Osteoporosis (Topic)	218313

3	Extracellular vesicles (Topic) OR Extracellular Vesicle (Topic) OR Vesicle, Extracellular (Topic) OR Vesicles, Extracellular (Topic) OR Exovesicles (Topic) OR Exovesicle (Topic) OR Apoptotic Bodies (Topic) OR Apoptotic Body (Topic) OR Bodies, Apoptotic (Topic) OR Body, Apoptotic (Topic) OR Exosomes (Topic) OR Cell-Derived Microparticles (Topic) OR Cell-Derived Microparticle (Topic) OR Cell Derived Microparticles (Topic) OR Microparticle, Cell-Derived (Topic) OR Shedding Microvesicles (Topic) OR Microvesicle, Shedding (Topic) OR Microvesicles, Shedding (Topic) OR Shedding Microvesicle (Topic) OR Cell Membrane Microparticles (Topic) OR Cell Membrane Microparticle (Topic) OR Membrane Microparticle, Cell (Topic) OR Membrane Microparticles, Cell (Topic) OR Microparticle, Cell Membrane (Topic) OR Microparticles, Cell Membrane (Topic) OR Microparticles, Cell-Derived (Topic) OR Microparticles, Cell Derived (Topic) OR Ectosomes (Topic) OR Ectosome (Topic) OR Circulating Cell-Derived Microparticles (Topic) OR Cell-Derived Microparticle, Circulating (Topic) OR Cell-Derived Microparticles, Circulating (Topic) OR Circulating Cell-Derived Microparticle (Topic) OR Circulating Cell Derived Microparticles (Topic) OR Microparticle, Circulating Cell-Derived (Topic) OR Microparticles, Circulating Cell-Derived (Topic)	134961
4	#1 AND #2 AND #3	561

Table S2. Summary of included studies: EV sources, isolation and characterization methods, modification strategies, and major *in vitro* findings.

Reference	Study	EV	Parent cells	Isolation	Characterization	Engineering of EVs	*In vitro* results
21	Cui, 2022	Exosomes	Human iPSC-MSCs	UC	NTA; DLS; TEM	EVs were conjugated with a bone targeting peptide SDSSD; si*Shn3* was loaded into EVs through electroporation	The SDSSD peptide could enhance osteoblast uptake;<br>Inhibits osteoclastogenesis;<br>Promotes osteogenesis and angiogenesis.
22	Lei, 2025	Exosomes	Human UC-MSCs	UC	NTA, SEM	Not mentioned.	Functionalized “cell climbing frame” facilitates aging BMSC recruitment, adhesion and migration; rejuvenates aging BMSC viability and regulate surrounding microenvironment; enhances osteogenic differentiation of aging BMSCs

23	Qu, 2024	apoVs	Human ESCs	Differential centrifugation	NTA; TEM; FC	Not mentioned.	Decrease the levels of cellular senescence markers and increase the level of proliferation markers in aging BMSCs. Promote osteogenic differentiation and reduce adipogenic differentiation ability of aging BMSCs. Decrease mtDNA release from aging BMSCs.
24	Xue, 2024	apoVs	Mouse BMSCs	Differential centrifugation	TEM; NTA; WB; SIM	Not mentioned.	Decrease the expression of senescence markers; Rescue apoptotic resistance; Restore intracellular Ca^{2+} in BMSCs under microgravity.
25	Yang, 2024	apoVs	Human DPSCs	Differential centrifugation	TEM; NTA; WB	Not mentioned.	Promote osteogenic differentiation of BMSCs.
26	Zhu, 2023	apoVs	Human BMSCs	Differential centrifugation	TEM; NTA; FC; WB	Not mentioned.	Promote osteogenic differentiation of BMSCs. Inhibit osteoclast formation of RAW264.7 Cells.

27	Lei, 2022	apoVs	Mouse BMSCs	Differential centrifugation	TEM; NTA; FC; WB.	Not mentioned.	Young BMSC-derived apoVs could restore the self-renewal capacity, rescue the imbalanced lineage differentiation fate and rejuvenate senescent phenotype of aged BMSCs.
28	Luo, 2019	Exosomes	Mouse mesenchymal stromal cells	ExoQuick-TC exosome precipitation kit	TEM; DLS; WB	EV surface was conjugated with BMSC-specific aptamers	Enhance the osteogenic activities of BMSCs. The conjugated aptamer facilitates the internalization efficacy of exosomes in BMSCs.
29	Zuo, 2019	Exosomes	Rat BMSCs	UC	TEM; WB	Not mentioned.	Accelerate DNA repair, alleviate radiation-induced oxidative stress, rescue the inhibition of proliferation, decrease the senescence-associated protein expression, restore the differentiation potential of irradiated BMSCs.
30	Zhang, 2024	Exosomes	Human BMSCs	UC	TEM; NTA; WB	Not mentioned.	Promote osteogenic differentiation of

							osteoblasts by promoting M2 macrophage polarization.
31	Han, 2023	Exosomes	Mouse BMSCs	UC	TEM; WB	EV surface was conjugated with BMSC-specific aptamers.	Normal BMSC-EVs promote osteogenesis and inhibit adipogenesis in BMSCs.
32	Wang, 2020	Exosomes	Mouse MSCs	UC	TEM; DLS	EVs were combined with alendronate by click chemistry.	Promote proliferation and osteogenesis in MSCs.
33	Gui, 2023	apoVs	Mouse BMSCs	Differential centrifugation	TEM; DLS; FC; WB	ApoVs were conjugated with bone-targeting peptide. $(DSS)_6$;<br>BMSCs were loaded with RNF146 via adenovirus transduction.	Promote osteogenic differentiation of BMSCs.
34	Qiu, 2021	Exosomes	Rat BMSCs	exoEasy Maxi Kit	TEM; WB	BMSCs transfected with miR-150-3p.	Promote osteogenic differentiation, proliferation and suppress apoptosis of osteoblasts.
35	Liu, 2018	apoVs	Mouse BMSCs	Differential centrifugation	FC	Not mentioned.	Promote proliferation and osteogenic differentiation of impaired MSCs.

36	Xu, 2024	Exosomes	Rat BMSCs	UC	TEM; NTA; WB	EVs were conjugated to GMNPs via anti-CD63 antibodies that specifically bind to CD63 proteins on the EV surface.	Inhibit osteoclast differentiation in bone marrow-derived macrophages.
37	Qi, 2023	Exosomes	Rat BMSCs	UC	TEM; NTA; WB	Not mentioned.	Promote proliferation and osteogenic differentiation of MG-63 cells;<br>Increase the proportion of cells in the G2 + S phase and decrease the proportion of cells in the G1 phase.
38	Behera, 2021	Exosomes	Mouse BMSCs	UC; total Exosome isolation reagent kit	WB; DLS	Not mentioned.	Promote proliferation, migration and tube formation of endothelial cells;<br>Promote proliferation and osteogenic differentiation of impaired BMSCs.
39	Li, 2021	Exosomes	Human BMSCs	exoEasy Maxi Kit	TEM; DLS; WB	Not mentioned.	Promote proliferation and osteogenic differentiation of BMSCs from OVX rats.

40	Xiao, 2021	Exosomes	Mouse BMSCs	UC	TEM; NTA; WB	Cyclic mechanical stretch-treated BMSCs and normal static-cultured BMSCs.	Inhibit osteoclastogenesis and actin ring formation in bone marrow-derived macrophages.
41	Sheng, 2022	Exosomes	Human ASCs	UC	TEM; NTA; WB	ASCs cultured in OM.	OM-EVs promote osteogenic differentiation of OVX BMSCs
42	Hu, 2021	Exosomes	Human ASCs	UC	TEM; NTA; WB	ASC transfected with miR-21.	Not mentioned.
43	Zhang, 2021	Exosomes	Rat ASCs	UC	TEM; NTA; WB	Not mentioned.	Suppress high glucose-induced pro-inflammatory cytokines secretion of osteoclasts.
44	Yao, 2023	Exosomes	Rat ASCs	UC	TEM; NTA; WB	Not mentioned.	Attenuate Dex-induced apoptosis, suppress oxidative stress in MC3T3-E1 Cells; Restore osteogenic function of Dex-treated MC3T3-E1 Cells.
45	Zhang, 2022	Exosomes	Rat ASCs	UC	TEM; NTA; WB	ASCs transfected with miR-146a.	Suppress high glucose-induced pro-inflammatory cytokines of osteoclasts and decrease the bone resorption capacity of osteoclasts.

46	Huang, 2022	Exosomes	Human BMSCs	Exoquick reagent	TEM; NTA; WB	Artesunate-treated BMSCs.	Promote proliferation and osteogenic differentiation of hFOB1.19 cells.
47	Qi, 2016	Exosomes	Human iPSC-MSC	UC	Tunable resistive pulse sensing analysis; WB	Not mentioned.	Promote proliferation and osteogenic differentiation of OVX BMSCs.
48	Lee, 2021	Exosomes	Human ASCs	Multi-filtration system based on tangential flowfiltration (Minimate TFF System)	TEM; DLS; FC; WB	Not mentioned.	Promote migration of BMSCs;<br>Inhibit osteoclastogenesis in RAW264.7 cells.
49	Li, 2024	apoVs	Mouse BMSCs	Differential centrifugation	TEM; DLS; NTA; FC; WB	Not mentioned.	Promote the proliferation, migration, and osteogenic differentiation of BMSCs.
50	Wang, 2023	Exosomes	Mouse BMSCs	UC	TEM; NTA; WB	Not mentioned.	Promote the osteogenic differentiation of MC-3T3 cells; Inhibit osteoclastic differentiation of RAW264.7 cells.
51	Sonoda, 2021	Exosomes	Human SHEDs	exoEasy Maxi kit	TEM; NTA; FC; WB	Not mentioned.	Promote osteogenic differentiation of BMSCs; Upregulate telomerase activity of BMSCs.
52	Huang, 2021	Exosomes	Rat BMSCs	UC	TEM; NTA; WB	BMSCs transfected with the GPNMB	GPNMB-EVs promote the proliferation and

						overexpression vector.	osteogenic differentiation of BMSCs
53	Rao, 2024	Exosomes	Human USCs	Optiprep density gradient ultracentrifugation	TEM; DLS; WB	Not mentioned.	Protect against serum deprivation- or hydrogen peroxide-induced cellular senescence and apoptosis of BMSCs.
54	Chen, 2019	Exosomes	Human USCs	ExoQuick-TC exosome precipitation kit	TEM; WB	Not mentioned.	Promote osteogenic differentiation of BMSCs; Inhibit osteoclastogenesis in RAW264.7 cells.
55	Liu, 2025	microvesicles	Human SHED cell aggregate	Differential centrifugation	TEM; NTA; WB	Not mentioned.	Promote proliferation and osteoblastic differentiation of aging BMSCs
56	Xu, 2024	Exosomes	Rat BMSCs	UC	TEM; NTA; WB	BMSCs transfected with over expressing plasmid-MEG3; EVs were conjugated to GMNPs via anti-CD63 antibodies that specifically bind to CD63 proteins on the EV surface.	Promote proliferation and osteogenic differentiation of osteoblasts.
57	Gong, 2020	Exosomes	Human ESCs	UC	TEM; qNano; WB	Not mentioned.	Ameliorate the senescent phenotypes, and promote

							the proliferation and osteogenic differentiation of BMSCs from SAMP8 mice.
58	Zhao, 2025	Exosomes	Human UCMSCs	UC	TEM; NTA; WB	Not mentioned.	Promote the proliferation and osteogenic differentiation of dexamethasone-treated BMSCs.
59	Hu, 2020	Exosomes	Human UCMSCs	Exoquick-TC Exosome precipitation kit	TEM; DLS; WB	Not mentioned.	Promote osteogenic differentiation and inhibit adipogenic differentiation of BMSCs; Inhibit osteoclastogenesis in RAW264.7 cells.
60	Wang, 2022	Exosomes	Mouse BMSCs	UC	TEM; NTA; FC	Not mentioned.	Promote osteogenic differentiation of MC3T3-E1 cells; Inhibit bone marrow macrophage differentiation into osteoclasts.
61	Huang, 2024	Exosomes	Human ESCs	UC	TEM; NTA; WB	Not mentioned.	Promote osteogenic differentiation of UCMSCs.
62	Li, 2021	Exosomes	BMSCs	UC	TEM; NTA; WB	EPI-preconditioned BMSCs	EPI-EVs promote proliferation and

							osteogenic differentiation of osteoblasts.
63	Li ,2021	Exosomes	Rat BMSCs	UC	TEM; NTA; WB	PT-preconditioned BMSCs	PT-EVs promote osteogenic differentiation of pre-osteoblasts.
64	Liu, 2021	Exosomes	Human BMSCs	UC	TEM; NTA; WB	BMSC transfected with miR-20a.	Promote the migration and osteogenesis of hBMSCs.
65	Lu, 2021	Exosomes	Human WJMSCs	UC<br>Exo-Prep kit	TEM; NTA; WB	Not mentioned.	Promote osteogenic differentiation of BMSCs.
66	Chang, 2024	Exosomes	Mouse BMSCs	UC	TEM; DLS; WB	BMSCs transfected with RANK.	Suppress osteoclast differentiation;<br>Promote the osteogenic differentiation of MSCs.
67	Ge, 2021	Exosomes	Human UCMSCs	UC	TEM; NTA; WB	Osteogenically induced UCMSCs.	Promote proliferation and osteogenic differentiation of osteoblasts.
68	Wang, 2025	Exosomes	Mouse BMSCs	UC	TEM; NTA; WB	Not mentioned.	Promote cell proliferation of VPS33B-deficient MSCs and profoundly ameliorate cellular senescence.
69	Tang, 2025	Exosomes	Mouse BMSCs	UC	TEM; NTA; WB	Not mentioned.	Promote osteogenic differentiation and inhibit adipogenic differentiation of BMSCs.

70	Li, 2026	Exosomes	Mouse BMSCs	UC	TEM; NTA; WB	BMSCs transfected with CXCR4; EVs electroporated with GSH.	Promote proliferation and osteogenic differentiation of aged BMSCs; Suppress oxidative stress, improve mitochondrial function and delay aging in aged BMSCs.
71	Jiang, 2026	ApoVs	Mouse BMSCs	Differential centrifugation	Cryo-electron microscopy; NTA; Nano-flow cytometry	Engineered Thy1-enriched apoVs produced through magnetic-activated sorting and protein corona technology.	Promote osteogenic differentiation and inhibit adipogenic differentiation of aged MSCs.
72	Wang, 2026	Exosomes	Mouse BMSCs	ExoQuick-TC kit	TEM; NTA; WB	Not mentioned.	Promote osteogenic differentiation of MC3T3-E1 cells; Suppresses Th17 cell differentiation and enhances Treg cell generation.
73	Liu, 2026	Exosomes	Human BMSCs	UC	TEM; NTA; WB	Not mentioned.	Promote osteogenic differentiation of hFOB1.19 cells.
74	Zhai, 2024	Exosomes	Mouse BMSCs	Total Exosome Isolation reagent	TEM; NTA; WB	Exosomes were modified with BMSC-affinity peptide E7 by lentivirus packaging	COR alleviates oxidative stress and cellular senescence of BMSCs.

						techniques and then loaded with COR by both incubation and sonication methods.	
75	Long, 2025	Exosomes	Mouse BMSCs	UC	TEM; NTA; FC	BMSCs pretreated with SBG.	Promote proliferation and osteogenic differentiation of MC3T3-E1 cells.
76	Zhao, 2025	Exosomes	Rat BMSCs	ExoQuick Exosome Rapid Extraction Kit	TEM; NTA; WB	BMSCs transfected with miR-133b-3p mimic.	Promote TGF-β1 expression and $CD4^{+}CD25^{+}Foxp3^{+}$ Treg cell differentiation.
77	Ning, 2026	Exosomes	Human BMSCs	UC	NTA; WB	BMSCs pretreated with IBA.	Promote proliferation and osteogenic differentiation of hFOB1.19 cells.
78	Li, 2025	Exosomes	Rat ASCs	UC	TEM; NTA; WB	Not mentioned.	Promote proliferation and osteogenic differentiation of OVX BMSCs; Decrease ROS production in OVX BMSCs.
79	Hao, 2025	Exosomes	ABPCs	UC	TEM; NTA; TRPS; WB	Not mentioned.	Promote proliferation and osteogenic differentiation of aged BMSCs; Attenuate phenotypes of senescence in aged BMSCs.

80	He, 2025	Exosomes	BMSCs from osteoporosis patients	UC	TEM; NTA; WB	Not mentioned.	Inhibit tube formation of HUVECs and osteogenic differentiation of hFOB1.19 cells.
81	Wang, 2025	Exosomes	Human BMSCs	UC	TEM; NTA; WB	BMSCs transfected with miR-20b.	Promote osteogenic differentiation and mitochondrial function of OVX osteoblasts.
82	Kim, 2025	Exosomes	Human AMSCs	Ultrafiltration	TEM; NTA; WB	Hypoxia (2% O_2) pretreated AMSCs.	Promote proliferation of MC3T3-E1 cells; Protect MC3T3-E1 cells against oxidative stress and hypoxic injury.
83	Cheng, 2024	apoVs	Human BMSCs	Differential centrifugation	TEM; NTA; WB	MSCs transfected with RNA oligo to knockdown hsa-miR-4485-3p.	Enhance osteogenic differentiation and suppress adipogenic differentiation of MSCs.
84	Tang, 2025	Exosomes	Mouse BMSCs	UC	TEM; NTA; WB	BMSCs pretreated with f-CA(OH) nanomaterials.	Promote macrophage polarization towards M2 phenotype; Suppress osteoclast maturation and TRAP secretion; Promote osteogenic differentiation of MC3T3-E1 cells; Promote tube formation of endothelial cells.

Abbreviations: EVs: extracellular vesicles; iPSC-MSCs: induced pluripotent stem cell-derived mesenchymal stem cells; UC: ultracentrifugation; NTA: nanoparticle tracking analysis; DLS: dynamic light scattering; TEM: transmission electron microscopy; WB: western blotting; UC-MSC: umbilical cord-derived mesenchymal stem cells; apoVs: apoptotic vesicles; ESCs: embryonic stem cells; FC: flow cytometry; BMSCs: bone marrow-derived mesenchymal stem cells; SIM: Super-resolution structured illumination microscopy; DPSCs: human dental pulp stem cells; GMNP: polyethylene glycol-modified magnetic nanoparticles; ASCs: adipose-derived mesenchymal stem cells; OM: osteogenic induction medium; OVX: ovariectomy; SHEDs: stem cells from human exfoliated deciduous teeth; USCs: urine-derived stem cells; EPI: epimedium; PT: plastrum testudines; WJMSCs: wharton's jelly-derived mesenchymal stem cells; GSH: glutathione; COR: cordycepin; SBG: scutellaria baicalensis georgi; IBA: isobavachin; ABPCs: antler blastema progenitor cells; AMSCs: human amniotic membrane stem cells.

Table S3. Summary of included studies: animal models, evaluation of EV safety and *in vivo* biodistribution, routes of EV administration and dosages, major *in vivo* findings, and underlying molecular mechanisms.

References	Study	Animal model	Safety evaluation	*In vivo* biodistribution evaluation	Method of administration	*In vivo* results	Potential molecular mechanism
21	Cui, 2022	Mouse model of OVX-induced osteoporosis.	Histologic analysis of major organs (heart, liver, spleen, lung, brain and kidney).	Yes.	Intravenous infusion of EVs (1.0×10^{10}) once a week for 6 weeks.	Prevent OVX-induced bone loss (increase BV/TV and Tb. N, decrease Tb. Sp), inhibit osteoclastogenesis and promotes type H vessel formation.	Through highly expressed osteogenic miRNAs, osteoclast inhibitory miRNAs and proangiogenic miRNAs.
22	Lei, 2025	Femoral condyle defect model in OVX-induced osteoporotic rats.	Histologic analysis of major organs (heart, liver, spleen, lung, and kidney).	Not mentioned.	Different groups of scaffolds (50 μg EVs/scafffod) were randomly implanted into the osteoporotic bone defect of the rats.	Promotes osteoporotic bone regeneration and integration (increase BV/TV and BS/TV).	Through upregulation of genes involved in bone formation and downregulation of genes associated with bone resorption.
23	Qu, 2024	Mouse model of senile	Not mentioned.	Not mentioned.	Intravenous infusion of ESC-	Increase BV/TV, BMD and Tb.n in the	ESC-apoVs inherit TCOF1 to rejuvenate aging BMSCs and

		osteoporosis.			apoVs (1.6 × 10^{10}) twice a week for two consecutive months.	femur of aging mice; Increase the number of ALP+ osteoblasts and decrease the number of TRAP+ osteoclasts in the femur of aging mice; Decrease aging markers and increase proliferation markers in the bone marrow.	rescue senile osteoporosis via FLVCR1-mediated mitochondrial functional homeostasis。
24	Xue, 2024	Mouse model of HU-induced disuse osteoporosis.	Not mentioned.	Not mentioned.	Intravenous infusion of apoVs (5 × 10^9) twice a week for 2 weeks.	Ameliorate osteopenia phenotype in HU mice (increase BMD and BV/TV); Reduce the number of senescent cells in unloading bone tissue.	Piezo1 carried by apoVs rescues impaired calcium influx and apoptotic resistance induced by mechanical unloading, thereby rescuing cellular apoptotic resistance and senescent cell accumulation.
25	Yang, 2024	Mouse model of OVX-induced osteoporosis; Mouse model of senile osteoporosis.	Histologic analysis of heart, liver, spleen, lung, and kidney.	Yes.	Tail vein injection (dosage not mentioned) once a week for 8 weeks.	Reduce the loss of microarchitecture and bone mass (increase Tb. BMD, BV/TV, Tb. Th, and Tb. N, decrease Tb. Sp) in both OVX and aged	Promote osteogenic differentiation of MSCs through the ERK1/2 signaling pathway.

						mice.	
26	Zhu, 2023	Mouse model of OVX-induced osteoporosis; Mouse model of senile osteoporosis.	Histologic analysis of mouse viscera.	Yes.	Tail vein injection (20 μg apoVs per 30 g weight) once a week for 2 months.	Attenuate bone loss (increase BMD, BV/TV, Tb. Th, and Tb. N, decrease Tb. Sp) in both OVX and aged mice.	Through delivery of miR1324, which inhibit expression of the target gene SNX14 and thus activate the SMAD1/5 pathway in target cells.
27	Lei, 2022	Mouse model of senile osteoporosis.	Not mentioned.	Yes.	Tail vein injection (20 μg apoVs) semimonthly for 6 months.	ApoVs infusion rescue age-related bone-fat imbalance in the skeleton of aged mice (increase BMD, BV/TV and ratio of trabecular bone; diminish the number and area of adipocytes in the bone marrow). Recover nuclear abnormalities and rejuvenate senescent MSCs from aged mice.	Apoptotic MSCs enrich Rab7 to apoVs to promote autolysosome formation and autophagy activation of recipient aged MSCs.

28	Luo, 2019	Mouse model of OVX-induced osteoporosis	Not mentioned.	Yes.	Tail vein injection (100 μg EVs) once a week for 2 months.	Ameliorates osteoporosis (increase BV/TV, Tb. Th, and Tb. N) in OVX mice.	Through delivery of enriched miR-26a.
29	Zuo, 2019	Rat model of irradiation-induced osteoporosis.	Not mentioned.	Not mentioned.	Tail vein injection (1.6 mg/kg EVs).	Alleviate bone loss induced by irradiation (increase BV/TV, Tb. N, Tb.Th and Conn.D)	Activate the wnt/β-catenin pathway of BMSCs after irradiation.
30	Zhang, 2024	Mouse model of OVX-induced osteoporosis.	Not mentioned.	Not mentioned.	Intra-femoral injection (20 mg EVs) twice per week.	Attenuate bone loss in mice caused by OVX (increase BV/TV, Tb. N and Tb.Th, decrease Tb. Sp).	Upregulate TRIM25 expression, which facilitated the ubiquitination and degradation of TREM1.
31	Han, 2023	Mouse model of diabetic osteoporosis; Femoral bone injury model in diabetic mice.	Not mentioned.	Yes.	Tail vein injection (100 μg EVs) twice a week.	Alleviated bone-fat imbalance in diabetic mice (increase Tb. BV/TV and Tb. N, decrease Tb. Sp); Promote bone regeneration in diabetic mice.	MiR-221, as one of the highly expressed miRNAs within diabetic BMSCs-Exos, showed abilities of suppressing osteogenesis and promoting adipogenesis both *in vitro* and *in vivo*. MiR-221 regulates osteogenesis and

							adipogenesis by direct targeting RUNX2.
32	Wang, 2020	Rat model of OVX-induced osteoporosis.	Histologic analysis of heart, kidney and liver; Serum analysis for heart and kidney toxicity.	Yes.	Tail vein injection (750 µg EVs) twice per week for a total of 16 injections.	Promote bone regeneration in osteoporotic conditions (increase BMD, BV/TV, Tb.N, Tb.Th, decrease Tb. Sp)	Not mentioned.
33	Gui, 2023	Mouse model of OVX-induced osteoporosis.	Histologic analysis of heart, liver, spleen, lungs, and kidneys; Serum analysis for heart, liver and kidney toxicity.	Yes.	Tail vein injection (10 mg/kg) once a week for four weeks.	Attenuate bone loss in mice caused by OVX (increase BMD, BV/TV, Tb.N and Tb.Th, decrease Tb. Sp).	RNF146, an E3 ubiquitin ligase with a ring domain, mediates Axin degradation, consequently activating the Wnt/β-catenin pathway to facilitate osteogenesis.
34	Qiu, 2021	Rat model of OVX-induced osteoporosis.	Not mentioned.	Not mentioned.	Caudal vein injection (100 µg EVs).	Attenuate the osteoporotic phenotype in femoral tissues of OVX rats	Through delivery of miR-150-3p.

						(increase BMD, BV/TV, Tb. N and Tb. Th, decrease Tb. Sp).	
35	Liu, 2018	Mouse model of OVX-induced osteoporosis.	Not mentioned.	Yes.	Tail vein injection (4×10^6 apoVs) once a week for 4 weeks.	Ameliorate osteopenia phenotype in OVX mice (increase BMD and BV/TV).	Through delivery of ubiquitin ligase RNF146 and miR-328-3p to inhibit Axin1 and thereby activate the Wnt/β-catenin pathway.
36	Xu, 2024	Rat model of diabetic osteoporosis	Not mentioned.	Yes.	Tail vein injection (400 µL of nanoparticles/kg in PBS).	Alleviate osteoporosis in DO rats (increase BV/TV, Tb. N, Tb. Th and BMD).	Through delivery of miR-15b-5p to osteoclasts, downregulating GFAP expression and inhibiting osteoclast differentiation.
37	Qi, 2023	Rat model of OVX-induced osteoporosis.	Not mentioned.	Not mentioned.	Tail vein injection (100 µL EVs at the concentration of 1 mg/mL) once a week for 8 weeks.	Alleviate osteoporosis in OVX rats (increase BV/TV, Tb. N, and BMD, decrease Tb. Sp).	Through the ERK-ERα signaling pathway.
38	Behera, 2021	Mouse model of metabolic osteoporosis (CBS deficiency).	Not mentioned.	Yes.	Tail vein injection (100 µg/mL EVs) three times/week for 8 weeks.	Alleviate osteoporosis in $CBS^{+/-}$ mice (increase BV/TV, Tb. N, Tb. Th and BMD,	Exosomal lnc-H19 acts as “sponges” to absorb miR-106 and regulate the expression of angiogenic factor, Angpt1 that activates lnc-H19/Tie2-

						decrease Tb. Sp).	NO signaling in mesenchymal and endothelial cells.
39	Li, 2021	Rat model of OVX-induced osteoporosis.	Not mentioned.	Not mentioned.	Tail vein injection (100 μL 10^{13}/mL EVs) once a week for 28 days.	Alleviate osteoporosis in OVX rats (increase BV/TV, Tb. N and Tb. Th, decrease Tb. Sp)	Transfer miR-186 to promote osteogenesis through the Hippo signaling pathway.
40	Xiao, 2021	Mouse model of HU-induced disuse osteoporosis.	Not mentioned	Not mentioned	Tail vein injection (5 mg/kg body weight, 0.1 mL per injection) twice a week for 4 weeks.	Alleviate osteoporosis in HU mice (increase BMD, BV/TV, Tb. Th, Tb. N and Ct. Th, decrease Tb. Sp).	CMS-Exosomes impair osteoclast differentiation via inhibition of the RANKL-induced NF-κB signaling pathway.
41	Sheng, 2022	Mouse model of OVX-induced osteoporosis.	Histologic analysis of heart, lung, spleen, kidneys and liver.	Yes.	Tail vein injection (100 μg EVs) every 3 days for a total of 8 injections.	Alleviate osteoporosis in OVX rats (increase BMD, BV/TV, Tb. N and Tb. Th, decrease Tb. Sp).	miR-335-3p play a negative regulatory role in the osteogenic differentiation of BMSCs by targeting Aplnr.
42	Hu, 2021	Mouse model of PGIA.	Not mentioned.	Yes.	Intraspinal injection (1.6 μg/μL, 0.5 μL) once a week for 8	Alleviate osteoporosis in PGIA-mice (increase BMD and bone	MiR-21 may act as a switch between inflammation and new bone formation in a TNF-α concentration-

					weeks.	mineral content in lumbar spine).	dependent manner in AS.
43	Zhang, 2021	Rat model of diabetic osteoporosis.	Not mentioned.	Not mentioned.	Tail vein injection (1.6 mg/kg EVs) every two days.	Alleviate osteoporosis in diabetic osteoporotic rats (increase BMD and bone mineral content).	By the suppressing of NLRP3 inflammasome activation in osteoclasts.
44	Yao, 2023	Rat model of GIOP.	Not mentioned.	Not mentioned.	Tail vein injection (50 or 100 µg EVs) three times a week for 5 weeks.	Alleviate osteoporosis in GIOP rats (increase BMD, BV/TV, Tb. Th and Tb. N, decrease Tb. Sp).	By activating the Nrf2/HO-1 signaling pathway.
45	Zhang, 2022	Rat model of diabetic osteoporosis.	Not mentioned.	Not mentioned.	Tail vein injection (1.6 mg/kg EVs) every two days.	Alleviate osteoporosis in diabetic osteoporotic rats (increase BMD and bone mineral content);<br>Suppress the activation of inflammasome of osteoclasts.	MiR-146a was predicted to base-pair with the TRAF6 and IRAK1 in their 3'UTR regions.

46	Huang, 2022	Mouse model of OVX-induced osteoporosis	Not mentioned.	Not mentioned.	Intra-femoral bone marrow injection (20 μL EVs) twice a week.	Alleviate osteoporosis in OVX mice (increase BMD, BV/TV, Tb. Th and Tb. N, decrease Tb. Sp).	By transfer of long noncoding RNA SNHG7 to regulate TAF15/RUNX2 pathway.
47	Qi, 2016	Calvarial bone defect model in OVX-induced osteoporotic rats	Not mentioned.	Not mentioned.	β-TCP scaffolds + 100 μg/200 μg EVs were implanted into the osteoporotic bone defects of the rats.	Promote bone regeneration (increase BMD and BV/TV) and neovascularization.	Not mentioned.
48	Lee, 2021	Mouse model of OVX-induced osteoporosis.	Not mentioned.	Yes.	Tail vein injection (1 or 5×10^8 particles/100 μL PBS) thrice a week for 2 weeks.	Alleviate osteoporosis in OVX mice (increase BMD, BV, BV/TV, Tb. Th and Tb. N)	OPG is critical for the therapeutic effects of ASC-EVs; miR-21-5p in ASC-EVs inhibit osteoclast differentiation through Acvr2a down-regulation; let-7b-5p in ASC-EVs significantly reduced the expression of genes related to osteoclastogenesis.
49	Li, 2024	Mouse model of OVX-induced osteoporosis.	Not mentioned.	Yes.	Tail vein injection (100 μg apoVs) once a	Alleviate osteoporosis in OVX mice (increase BV,	By transfer of Ras to activate the Ras/Raf1/Mek/Erk pathway.

					week for 2 months.	BV/TV and Tb. N, decrease Tb. Sp).	
50	Wang, 2023	Mouse model of OVX-induced osteoporosis.	Not mentioned.	Not mentioned.	Tail vein injection (100 µg EVs) twice a week for 8 weeks.	Alleviate osteoporosis in OVX mice (increase BMD, BV/TV, Tb. Th and Tb. N, decrease Tb. Sp).	By transfer of USP7 to stabilize YAP1 protein through deubiquitination modification; YAP1 activates the Wnt/β-catenin pathway.
51	Sonoda, 2021	Mouse model of OVX-induced osteoporosis.	Not mentioned.	Yes.	Tail vein injection (100 µg EVs in 100 µL PBS) for 4 weeks.	Alleviate osteoporosis in OVX mice (increase BMD, BV/TV, Tb. Th and Tb. N).	By rescuing the mRNA levels of TERT, telomerase activity and and SEMA3A secretion.<br>EV-contained miR-346 may epigenetically regulate TERT mRNA expression in recipient BMSCs.
52	Huang, 2021	Rat model of OVX-induced osteoporosis.	Histologic analysis of rain, heart, lungs, spleen, and kidneys; Evaluation of inflammatory responses.	Not mentioned.	Caudal vein injection (100 µg EVs) once a week for 8 weeks	Alleviate osteoporosis in OVX rats (increase BMD, BV/TV, Tb. Th and Tb. N, decrease Tb. Sp).	Through activation of Wnt/β-catenin signaling.

53	Rao, 2024	Mouse model of senile osteoporosis (senescence-accelerated mice (SAMP8) and natural aging mice)	Not mentioned.	Yes.	Tail vein injection (100 µg EVs) once a week for 2 months in SAMP8 mice and 3 months in natural aging mice.	Alleviate osteoporosis in SAMP8 mice (increase BV/TV, Tb. Th, and Ct. Th); Alleviate osteoporosis in natural aging mice (increase BV/TV, Tb. Th, Tb. N, and Ct. Th, decrease Tb. Sp).	By transferring PLAU and TIMP1 proteins to inhibit matrix metalloproteinases, $P16^{INK4}$ and $P21^{cip1}$.
54	Chen, 2019	Mouse model of OVX-induced osteoporosis; Mouse model of senile osteoporosis; Mouse model of disuse osteoporosis	Histologic analysis of spleen and serum inflammatory cytokines detection.	Yes.	Tail vein injection (100 µg EVs) once a week for 2 months in OVX-induced osteoporotic mice, 3 months in senile osteoporotic mice and 3 weeks in disuse osteoporotic mice.	Alleviate osteoporosis in OVX mice (increase BV/TV, Tb. Th, Tb. N, Es. Pm and Ct. Th, decrease Tb. Sp). Enhance trabecular and cortical bone mass and bone strength in aging and hind-limb unloading-induced osteoporotic mice.	By transferring CTHRC1 and OPG.

55	Liu, 2025	Mouse model of senile osteoporosis	Not mentioned.	Yes.	Tail vein injection (60 μg EVs) once a week for a total of 6 injections.	Alleviate osteoporosis in aging mice (increase BV/TV, Tb. N).	Rescues Golgi dysfunction and empowers senescent MSCs through transfer of the Golgi regulatory protein Syntaxin 5.
56	Xu, 2024	Rat model of diabetic osteoporosis	Not mentioned.	Yes.	Tail vein injection (400 μL of nanoparticles containing 10 mg/kg in PBS).	GMNPE-EVs-MEG3 alleviate osteoporosis in diabetic rats (increase BMD, BV/TV, Tb. N and Tb. Th).	Through the delivery of MEG3 to inhibit miR-3064-5p, activating NR4A3/PINK1/Parkin pathway, thereby enhance osteoblast proliferation and differentiation.
57	Gong, 2020	Mouse model of senile osteoporosis (SAMP8)	Not mentioned.	Yes.	Gavage treated every other day for 6 months with EVs (1 × 10^{10} particles per mouse, dissolved in PBS).	Alleviate osteoporosis in SAMP8 mice (increase BMD, BV/TV and Tb. N, decrease Tb. Sp).	Bioinformatics analyses predict that the protein components in the ESCs-EVs function in a synergistic way to induce the activation of several canonical signaling pathways, including Wnt, Sirtuin, AMPK, PTEN signaling.
58	Zhao, 2025	Mouse model of GIOP	Not mentioned.	Not mentioned.	Intramuscular injection (100 μg EVs) every 2	Alleviate osteoporosis in GIOP mice (increase	By activating PI3K/AKT pathway-mediated ferroptosis inhibition.

					days for 8 weeks.	BV/TV, Tb. Th and Tb. N, decrease Tb. Sp).	
59	Hu, 2020	Mouse model of OVX-induced osteoporosis; Mouse model of senile osteoporosis; Mouse model of disuse osteoporosis	Not mentioned.	Not mentioned.	Tail vein injection (100 μg EVs) once a week for 2 months (OVX model); 100 μg EVs once a week for 3 months (aged model); 100 μg EVs twice a week for 21 days (disuse model).	Alleviate osteoporosis in OVX mice (increase BV/TV, Tb. Th and Ct. Th); No significant alleviation of osteoporosis in aged model; Alleviate osteoporosis in disuse model (increase BV/TV, Tb. Th, Tb. N and Ct. Th).	By delivering CLEC11A.
60	Wang, 2022	Mouse model of OVX-induced osteoporosis	Not mentioned.	Not mentioned.	EVs were injected through the periosteum of the femur into mice twice a week for 1 week.	Alleviate osteoporosis in OVX mice (increase BMD, BV/TV, Tb. Th and Tb. N, decrease Tb. Sp).	Through the miR-27a/DKK2/Wnt/β-catenin signaling pathway.
61	Huang, 2024	Knee joint bone defect model in OVX-induced	Not mentioned.	Not mentioned.	Chi/ColI scaffolds loaded with ESC-EVs	Promote bone regeneration and increase BMD,	By delivering enriched miR-21-5p as a key regulator that targets YAP1 and activates

		osteoporotic rats			(200 μg) were implanted into the bone defects.	BV/TV, Tb. Th and Tb.N.	the Wnt/β-catenin signaling pathway.
62	Li, 2021	Rat model of OVX-induced osteoporosis	Not mentioned.	Not mentioned.	Tail vein injection (100 ng/mL EVs) for 12 weeks.	Alleviate osteoporosis in OVX rats (increase BMD and BV/TV).	By delivering enriched miR-27a-5p to suppress Atg4B, an important factor involved in the process of autophagy.
63	Li ,2021	Rat model of OVX-induced osteoporosis	Not mentioned.	Not mentioned.	Tail vein injection (100 ng/100g/d EVs) for 10 weeks.	Alleviate osteoporosis in OVX rats (increase BMD, BV/TV and Tb.N, decrease Tb. Sp).	Dramatically down-regulated miR-330-5p in PT-EVs negatively regulate osteogenesis by targeting Tnc to control Wnt/β-catenin signaling.
64	Liu, 2021	Femoral defect model in OVX-induced osteoporotic rats	Not mentioned.	Not mentioned.	1.5 ml of sEV-20a solution (50 wt%) were mixed with HA solution and injected into the pores of porous titanium scaffolds.	Promote bone regeneration (increase BMD and BV/TV).	MiR-20a could enhance osteogenesis by targeting BAMBI.
65	Lu, 2021	Mouse model of OVX-induced osteoporosis	Not mentioned.	Yes.	Tail vein injection (100 μg or 200 μg EVs)	Alleviate osteoporosis in OVX rats (increase Tb.	WJMSC- EV-miRNAs, including mir-21, mir-29, and mir-221, potentially mediate

					once a week for 2 months.	BMD, BV/TV, Tb. Th and Tb. N, decrease Tb. Sp).	the curative effect of osteoporosis by targeting the PI3K/AKT signaling pathway.
66	Chang, 2024	Mouse model of OVX-induced osteoporosis	Histologic analysis of hearts, livers, spleens, lungs and Kidneys.	Yes.	Tail vein injection (5 μg/μL EVs) every 3 days for 8 weeks.	Alleviate osteoporosis in OVX mice (increase Tb. BMD, BV/TV and Tb. N, decrease Tb. Sp).	EVs effectively neutralize excess RANKL in bone tissue due to the RANK−RANKL interaction; MiRNA-21a-5p in EVs contributes to restoring bone metabolic homeostasis.
67	Ge, 2021	Mouse model of OVX-induced osteoporosis.	Not mentioned.	Not mentioned.	Intraperitoneal injection (0.5 mg/kg EVs) every 3 days for 6 weeks.	Alleviate osteoporosis in OVX mice (increase BMD, BV/TV, Tb. N, Conn. D, decrease Tb. Sp).	The expression levels of mir-2110 and mir-328-3p increase with prolonged osteogenic differentiation and regulate target genes associated with bone differentiation.
68	Wang, 2025	Mouse model of senile osteoporosis.	Not mentioned	Not mentioned	Intramedullary injection (50 μg EVs) on day 1 and day 14.	Alleviate osteoporosis in aged mice (increase BMD, BV/TV and Tb. N, decrease Tb. Sp).	MiR-136-3p and miR-146a-5p were highly enriched in Y-Exosomes, which promote cell proliferation while inhibiting inflammation by targeting the PI3K-Akt and NF-κB pathway, respectively。

69	Tang, 2025	Mouse model of OVX-induced osteoporosis.	Not mentioned	Not mentioned	Tail vein injection (100 μL of 10^{13}/ml EVs) once a week for 28 days.	Alleviate osteoporosis in OVX mice (increase BMD).	By delivery of lncRNA-SNHG14 to regulate osteogenic-adipogenic balance in BMSCs by mediating miR-27a-3p/LMNB1 axis.
70	Li, 2026	Mouse model of senile osteoporosis.	Histologic analysis of heart, liver, spleen, lung, and kidney; Hemolysis testing; Complete blood count analysis	Yes.	Tail vein injection.	Alleviate osteoporosis in OVX mice (increase BMD, BV/TV, Tb. N and Tb. Th, decrease Tb. Sp); Suppress oxidative stress; Improve mitochondrial function.	DNA methylation-mediated suppression of GSS represents an upstream lesion limiting endogenous GSH synthesis and supply in aged bone, thereby constraining osteoblast differentiation.
71	Jiang, 2026	Mouse model of senile osteoporosis.	Blood tests; blood biochemistry; body length; body weight; Organ tissue examinations.	Yes.	Tail vein injection once a week.	1w apoVs and 3m apoVs alleviate osteoporosis in aged mice (increase BMD and BV/TV).	By activating the Thy1-ERK-TAZ signaling axis.
72	Wang, 2026	Mouse model of senile osteoporosis	Not mentioned.	Not mentioned.	Tail vein injection of 100 ng EVs once a	Alleviate osteoporosis in SAMP6 mice	By delivering miR-21-5p to modulate the SKP2/ubiquitination/FOXO1

		(senescence-accelerated prone mice SAMP6).			week.	(increase BMD, BV/TV, Tb. N and Tb. Th). Promote Treg cell differentiation and suppresses Th17 cell polarization in the bone marrow and the spleen.	signaling axis.
73	Liu, 2026	Rat model of OVX-induced osteoporosis.	Not mentioned.	Not mentioned.	Tail vein injection of 100 μL EVs (approximately 10^{13} particles/mL) once a week for 8 weeks.	Alleviate osteoporosis in OVX rats (increase BV/TV, Tb. N and Tb. Th, decrease Tb. Sp).	By delivering miR-509-5p to target SFRP1 and activate the Wnt/β-catenin signaling pathway.
74	Zhai, 2024	Mouse model of SFOP.	Histologic analysis of heart, liver, spleen, lung, and kidney.	Yes.	Tail vein injection	Alleviate osteoporosis in SFOP rats (increase BMD, BV/TV, Tb. N, Ct. Th and Tb. Th, decrease Tb. Sp).	By delivering COR to regulate the PI3K/Akt/FOXO3 signaling pathway.
75	Long, 2025	Mouse model of OVX-induced osteoporosis.	Not mentioned.	Not mentioned.	Intraperitoneal injection every 3 days for 8 weeks.	Alleviate osteoporosis in OVX mice (increase BMD,	By upregulating Semaphorin 3A.

						BV/TV and Tb. N).	
76	Zhao, 2025	Rat model of OVX-induced osteoporosis.	Not mentioned.	Not mentioned.	Tail vein injection (200 μg EVs) once a week for 4 weeks.	Alleviate osteoporosis in OVX rats (increase BMD, BV/TV, Tb. N, Tb. Th, and Ct. Th, decrease Tb. Sp); Decrease osteoclast marker enzyme TRAP, MMP-9, and Cathepsin K expression.	By delivering miR-133b-3p to target TGF-β1, acting via the TGF-β1/Treg-mediated immune pathway.
77	Ning, 2026	Rat model of OVX-induced osteoporosis.	Not mentioned.	Not mentioned.	Tail vein injection (100 μg EVs) once a week for 8 weeks.	Alleviate osteoporosis in OVX rats (increase BMD, BV/TV, Tb. N and Tb. Th, decrease Tb. Sp).	By delivering miR-127-3p, which inhibits KIF3B and activates the Wnt/β-catenin pathway.
78	Li, 2025	Rat model of OVX-induced osteoporosis.	Not mentioned.	Not mentioned.	Tail vein injection (100 μg EVs).	Alleviate osteoporosis in OVX rats (increase BMD, BV/TV, Tb. N and Tb. Th, decrease Tb. Sp).	By obstructing the nuclear export of has circ 0028877 and modulating the has circ 0028877/miR-4728-3p/PTCH1 axis.

79	Hao, 2025	Mouse model of senile osteoporosis; Rhesus macaque model of senile osteoporosis.	Complete blood counts; serum biochemistry (liver and kidney functions).	Yes.	Tail vein injection (40 µg EVs) three times weekly for 28 days; Intravenous administration (15 mg EVs) every two weeks for 20 weeks.	Alleviate osteoporosis in aged mice (increase BMD, BV/TV, Tb. N and Tb. Th, decrease Tb. Sp); Alleviate osteoporosis in aged rhesus macaques (increase BMD, BV/TV, Tb. N and Tb. Th).	By delivering Prkar2a.
80	He, 2025	Mouse model of OVX-induced osteoporosis.	Not mentioned.	Not mentioned.	Intravenous injection.	Exacerbate osteoporosis (decrease BMD, Tb. N and Tb. Th) and angiogenesis in OVX mice.	By delivering miR-422a to target KLK4.
81	Wang, 2025	Rat model of OVX-induced osteoporosis.	Not mentioned.	Not mentioned.	Intravenous injection (100 µL of EVs (10^{13}/mL)) once a week for 4 weeks.	Alleviate osteoporosis in OVX rats (increase BMD, BV/TV, Tb. N, Ct. Th and Tb. Th).	By delivering miR-20b to activate the SIRT1/BMP2 signaling.

82	Kim, 2025	Rat model of OVX-induced osteoporosis.	Not mentioned.	Not mentioned.	Intravenous injection ($1×10^8$, $3×10^8$ or $1×10^9$ particles/rat) once a week for 8 weeks.	Alleviate osteoporosis in OVX rats (increase BMD, BV/TV and Tb. N, decrease Tb. Sp).	EVs containing high concentrations of growth factors and neurotrophic factors.
83	Cheng, 2024	Mouse model of OVX-induced osteoporosis; Mouse model of senile osteoporosis.	Not mentioned.	Yes.	Tail vein injection (20 μg per 30 g body weight apoVs) once a week for 8 weeks.	Alleviate osteoporosis in OVX mice (increase BMD, BV/TV and Tb. N, decrease Tb. Sp); Alleviate osteoporosis in aged mice (increase BMD, BV/TV and Tb. N, decrease Tb. Sp).	MiR-4485-3p in apoVs suppress the osteogenic differentiation and promote adipogenic differentiation of MSCs by regulating the AKT signaling pathway.
84	Tang, 2025	Mouse model of OVX-induced osteoporosis	Histologic analysis of heart, lung, liver, spleen, and kidney; Detection of hepatic and renal metabolic parameters;	Yes.	Tail vein injection (150 μL of EVs (100 μg/mL)).	Alleviate osteoporosis in OVX mice (increase BMD, BV/TV and Tb. N, decrease Tb. Sp); Remodel inflammatory microenvironments and regulating macrophage	Suppress osteoclast differentiation by blocking PI3K/Akt signaling and enhance osteogenesis via SMAD2/RUNX2 activation.

	Hemolysis testing.	polarization.	

Abbreviations: OVX: ovariectomy; EVs: extracellular vesicles; BV/TV: bone volume/total volume; Tb. N: trabecular number; Tb. Sp: trabecular separation; BS/TV: bone surface/total volume; BMD: bone mineral density; ESCs: embryonic stem cells; apoVs: apoptotic vesicles; HU: hindlimb unloading; Tb. Th: trabecular thickness; BMSCs: bone marrow-derived mesenchymal stem cells; MSCs: mesenchymal stem cells; ALP: alkaline phosphatase; TRAP: tartrate-resistant acid phosphatase; Conn.D: connectivity density; CBS: cystathionine β-synthase; Ct. Th: cortical thickness; CMS: cyclic mechanical stretch; PGIA: proteoglycan-induced ankylosing spondylitis; AS: ankylosing spondylitis; GIOP: glucocorticoid-induced osteoporosis; β-TCP: β-tricalcium phosphate; OPG: osteoprotegerin; Es. Pm: endosteal perimeter; PT: plastrum testudines; WJMSC: wharton's jelly-derived mesenchymal stem cells; GSS: glutathione synthetase; GSH: glutathione; SFOP: spaceflight-induced osteoporosis; COR: cordycepin; MMP-9: matrix metalloproteinase-9; TGF-β1: transforming growth factor-β1.

Table S4. Risk of bias assessed using SYRCLE risk of bias assessment tool.

References	Study	Selection bias			Performance bias		Detection bias		Attrition bias	Reporting bias	Other
		Sequence generation	Baseline characteristics	Allocation concealment	Random housing	Blinding	Random outcome assessment	Blinding	Incomplete outcome data	Selective outcome reporting	Other sources of bias
21	Cui, 2022	Unclear Risk	Unclear Risk	Unclear Risk	Unclear Risk	Unclear Risk	Unclear Risk	Unclear Risk	Unclear Risk	Low Risk	Low Risk
22	Lei, 2025	Unclear Risk	Unclear Risk	Unclear Risk	Unclear Risk	Unclear Risk	Unclear Risk	Unclear Risk	Unclear Risk	Low Risk	Low Risk
23	Qu, 2024	Unclear Risk	Unclear Risk	Unclear Risk	Unclear Risk	Unclear Risk	Unclear Risk	Unclear Risk	Unclear Risk	Low Risk	Low Risk
24	Xue, 2024	Unclear Risk	Unclear Risk	Unclear Risk	Unclear Risk	Unclear Risk	Unclear Risk	Unclear Risk	Unclear Risk	Low Risk	Low Risk
25	Yang, 2024	Unclear Risk	Unclear Risk	Unclear Risk	Unclear Risk	Unclear Risk	Unclear Risk	Unclear Risk	Unclear Risk	Low Risk	Low Risk

26	Zhu, 2023	Unclear Risk	Unclear Risk	Unclear Risk	Unclear Risk	Unclear Risk	Unclear Risk	Unclear Risk	Unclear Risk	Low Risk	Low Risk
27	Lei, 2022	Unclear Risk	Unclear Risk	Unclear Risk	Unclear Risk	Unclear Risk	Unclear Risk	Unclear Risk	Unclear Risk	Low Risk	Low Risk
28	Luo, 2019	Unclear Risk	Unclear Risk	Unclear Risk	Unclear Risk	Unclear Risk	Unclear Risk	Unclear Risk	Unclear Risk	Low Risk	Low Risk
29	Zuo, 2019	Unclear Risk	Unclear Risk	Unclear Risk	Unclear Risk	Unclear Risk	Unclear Risk	Unclear Risk	Unclear Risk	Low Risk	Low Risk
30	Zhang, 2024	Unclear Risk	Unclear Risk	Unclear Risk	Unclear Risk	Unclear Risk	Unclear Risk	Unclear Risk	Unclear Risk	Low Risk	Low Risk
31	Han, 2023	Unclear Risk	Unclear Risk	Unclear Risk	Unclear Risk	Unclear Risk	Unclear Risk	Unclear Risk	Unclear Risk	Low Risk	Low Risk
32	Wang, 2020	Unclear Risk	Unclear Risk	Unclear Risk	Unclear Risk	Unclear Risk	Unclear Risk	Unclear Risk	Unclear Risk	Low Risk	Low Risk

33	Gui, 2023	Unclear Risk	Unclear Risk	Unclear Risk	Unclear Risk	Unclear Risk	Unclear Risk	Unclear Risk	Unclear Risk	Low Risk	Low Risk
34	Qiu, 2021	Unclear Risk	Unclear Risk	Unclear Risk	Unclear Risk	Unclear Risk	Unclear Risk	Unclear Risk	Unclear Risk	Low Risk	Low Risk
35	Liu, 2018	Unclear Risk	Unclear Risk	Unclear Risk	Unclear Risk	Unclear Risk	Unclear Risk	Unclear Risk	Unclear Risk	Low Risk	Low Risk
36	Xu, 2024	Unclear Risk	Unclear Risk	Unclear Risk	Unclear Risk	Unclear Risk	Unclear Risk	Unclear Risk	Unclear Risk	Low Risk	Low Risk
37	Qi, 2023	Unclear Risk	Unclear Risk	Unclear Risk	Unclear Risk	Unclear Risk	Unclear Risk	Unclear Risk	Unclear Risk	Low Risk	Low Risk
38	Behera, 2021	Unclear Risk	Unclear Risk	Unclear Risk	Unclear Risk	Unclear Risk	Unclear Risk	Unclear Risk	Unclear Risk	Low Risk	Low Risk
39	Li, 2021	Unclear Risk	Unclear Risk	Unclear Risk	Unclear Risk	Unclear Risk	Unclear Risk	Unclear Risk	Unclear Risk	Unclear Risk	Low Risk

40	Xiao, 2021	Unclear Risk	Unclear Risk	Unclear Risk	Unclear Risk	Unclear Risk	Unclear Risk	Unclear Risk	Unclear Risk	Low Risk	Low Risk
41	Sheng, 2022	Unclear Risk	Unclear Risk	Unclear Risk	Unclear Risk	Unclear Risk	Unclear Risk	Unclear Risk	Unclear Risk	Low Risk	Low Risk
42	Hu, 2021	Unclear Risk	Unclear Risk	Unclear Risk	Unclear Risk	Unclear Risk	Unclear Risk	Unclear Risk	Unclear Risk	Low Risk	Low Risk
43	Zhang, 2021	Unclear Risk	Unclear Risk	Unclear Risk	Unclear Risk	Unclear Risk	Unclear Risk	Unclear Risk	Unclear Risk	Low Risk	Low Risk
44	Yao, 2023	Unclear Risk	Unclear Risk	Unclear Risk	Unclear Risk	Unclear Risk	Unclear Risk	Unclear Risk	Unclear Risk	Low Risk	Low Risk
45	Zhang, 2022	Unclear Risk	Unclear Risk	Unclear Risk	Unclear Risk	Unclear Risk	Unclear Risk	Unclear Risk	Unclear Risk	Low Risk	Low Risk
46	Huang, 2022	Unclear Risk	Unclear Risk	Unclear Risk	Unclear Risk	Unclear Risk	Unclear Risk	Unclear Risk	Unclear Risk	Low Risk	Low Risk

47	Qi, 2016	Unclear Risk	Unclear Risk	Unclear Risk	Unclear Risk	Unclear Risk	Unclear Risk	Unclear Risk	Unclear Risk	Low Risk	Low Risk
48	Lee, 2021	Unclear Risk	Unclear Risk	Unclear Risk	Unclear Risk	Unclear Risk	Unclear Risk	Unclear Risk	Unclear Risk	Low Risk	Unclear Risk
49	Li, 2024	Unclear Risk	Unclear Risk	Unclear Risk	Unclear Risk	Unclear Risk	Unclear Risk	Unclear Risk	Unclear Risk	Low Risk	Low Risk
50	Wang, 2023	Unclear Risk	Unclear Risk	Unclear Risk	Unclear Risk	Unclear Risk	Unclear Risk	Unclear Risk	Unclear Risk	Low Risk	Low Risk
51	Sonoda, 2021	Unclear Risk	Unclear Risk	Unclear Risk	Unclear Risk	Unclear Risk	Unclear Risk	Unclear Risk	Unclear Risk	Low Risk	Low Risk
52	Huang, 2021	Unclear Risk	Unclear Risk	Unclear Risk	Unclear Risk	Unclear Risk	Unclear Risk	Unclear Risk	Unclear Risk	Low Risk	Low Risk
53	Rao, 2024	Unclear Risk	Unclear Risk	Unclear Risk	Unclear Risk	Unclear Risk	Unclear Risk	Unclear Risk	Unclear Risk	Low Risk	Low Risk

54	Chen, 2019	Unclear Risk	Unclear Risk	Unclear Risk	Unclear Risk	Unclear Risk	Unclear Risk	Low Risk	Low Risk	Low Risk	Low Risk
55	Liu, 2025	Unclear Risk	Unclear Risk	Unclear Risk	Unclear Risk	Unclear Risk	Unclear Risk	Unclear Risk	Unclear Risk	Low Risk	Low Risk
56	Xu, 2024	Unclear Risk	Unclear Risk	Unclear Risk	Unclear Risk	Unclear Risk	Unclear Risk	Unclear Risk	Unclear Risk	Low Risk	Low Risk
57	Gong, 2020	Unclear Risk	Unclear Risk	Unclear Risk	Unclear Risk	Unclear Risk	Unclear Risk	Low Risk	Unclear Risk	Low Risk	Low Risk
58	Zhao, 2025	Unclear Risk	Unclear Risk	Unclear Risk	Unclear Risk	Unclear Risk	Unclear Risk	Unclear Risk	Unclear Risk	Low Risk	Low Risk
59	Hu, 2020	Unclear Risk	Unclear Risk	Unclear Risk	Unclear Risk	Unclear Risk	Unclear Risk	Unclear Risk	Unclear Risk	Low Risk	Low Risk
60	Wang, 2022	Unclear Risk	Unclear Risk	Unclear Risk	Unclear Risk	Unclear Risk	Unclear Risk	Unclear Risk	Low Risk	Low Risk	Low Risk

61	Huang, 2024	Unclear Risk	Unclear Risk	Unclear Risk	Unclear Risk	Unclear Risk	Unclear Risk	Unclear Risk	Unclear Risk	Low Risk	Low Risk
62	Li, 2021	Unclear Risk	Unclear Risk	Unclear Risk	Unclear Risk	Unclear Risk	Unclear Risk	Unclear Risk	Unclear Risk	Low Risk	Low Risk
63	Li ,2021	Unclear Risk	Unclear Risk	Unclear Risk	Unclear Risk	Unclear Risk	Unclear Risk	Unclear Risk	Unclear Risk	Low Risk	Low Risk
64	Liu, 2021	Unclear Risk	Unclear Risk	Unclear Risk	Unclear Risk	Unclear Risk	Unclear Risk	Unclear Risk	Unclear Risk	Low Risk	Low Risk
65	Lu, 2021	Unclear Risk	Unclear Risk	Unclear Risk	Unclear Risk	Unclear Risk	Unclear Risk	Unclear Risk	Unclear Risk	Low Risk	Low Risk
66	Chang, 2024	Unclear Risk	Unclear Risk	Unclear Risk	Unclear Risk	Unclear Risk	Unclear Risk	Unclear Risk	Unclear Risk	Low Risk	Low Risk
67	Ge, 2021	Unclear Risk	Unclear Risk	Unclear Risk	Unclear Risk	Unclear Risk	Unclear Risk	Unclear Risk	Unclear Risk	Low Risk	Low Risk

68	Wang, 2025	Unclear Risk	Unclear Risk	Unclear Risk	Unclear Risk	Unclear Risk	Unclear Risk	Unclear Risk	Unclear Risk	Low Risk	Low Risk
69	Tang, 2025	Unclear Risk	Unclear Risk	Unclear Risk	Unclear Risk	Unclear Risk	Unclear Risk	Unclear Risk	Unclear Risk	Low Risk	Low Risk
70	Li, 2026	Unclear Risk	Unclear Risk	Unclear Risk	Unclear Risk	Unclear Risk	Unclear Risk	Unclear Risk	Unclear Risk	Low Risk	Low Risk
71	Jiang, 2026	Unclear Risk	Unclear Risk	Unclear Risk	Unclear Risk	Unclear Risk	Unclear Risk	Unclear Risk	Unclear Risk	Low Risk	Low Risk
72	Wang, 2026	Unclear Risk	Unclear Risk	Unclear Risk	Unclear Risk	Unclear Risk	Unclear Risk	Low Risk	Unclear Risk	Low Risk	Low Risk
73	Liu, 2026	Unclear Risk	Unclear Risk	Unclear Risk	Unclear Risk	Unclear Risk	Unclear Risk	Unclear Risk	Unclear Risk	Low Risk	Low Risk
74	Zhai, 2024	Unclear Risk	Unclear Risk	Unclear Risk	Unclear Risk	Unclear Risk	Unclear Risk	Unclear Risk	Unclear Risk	Low Risk	Low Risk

75	Long, 2025	Unclear Risk	Unclear Risk	Unclear Risk	Unclear Risk	Unclear Risk	Unclear Risk	Unclear Risk	Unclear Risk	Low Risk	Low Risk
76	Zhao, 2025	Low Risk	Unclear Risk	Unclear Risk	Unclear Risk	Unclear Risk	Unclear Risk	Unclear Risk	Unclear Risk	Low Risk	Low Risk
77	Ning, 2026	Unclear Risk	Unclear Risk	Unclear Risk	Unclear Risk	Unclear Risk	Unclear Risk	Unclear Risk	Unclear Risk	Low Risk	Low Risk
78	Li, 2025	Unclear Risk	Unclear Risk	Unclear Risk	Unclear Risk	Unclear Risk	Unclear Risk	Unclear Risk	Unclear Risk	Low Risk	Low Risk
79	Hao, 2025	Low Risk	Low Risk	Low Risk	Unclear Risk	Low Risk	Unclear Risk	Low Risk	Low Risk	Low Risk	Low Risk
80	He, 2025	Unclear Risk	Unclear Risk	Unclear Risk	Unclear Risk	Unclear Risk	Unclear Risk	Unclear Risk	Unclear Risk	Low Risk	Low Risk
81	Wang, 2025	Low Risk	Unclear Risk	Unclear Risk	Unclear Risk	Unclear Risk	Unclear Risk	Low Risk	Unclear Risk	Low Risk	Low Risk

82	Kim, 2025	Unclear Risk	Unclear Risk	Unclear Risk	Unclear Risk	Unclear Risk	Unclear Risk	Unclear Risk	Unclear Risk	Low Risk	Low Risk
83	Cheng, 2024	Unclear Risk	Unclear Risk	Unclear Risk	Unclear Risk	Unclear Risk	Unclear Risk	Low Risk	Unclear Risk	Low Risk	Low Risk
84	Tang, 2025	Unclear Risk	Unclear Risk	Unclear Risk	Unclear Risk	Unclear Risk	Unclear Risk	Unclear Risk	Unclear Risk	Low Risk	Low Risk

Table S5. Comparison of the present review with previous systematic reviews and meta-analyses of stem cell-derived EVs for osteoporosis.

References	Study	Review type	Included studies	EV subtypes and sources	EV engineering strategies	Molecular mechanisms
9	He, 2023	Systematic review and meta-analysis	11	Stem cell-derived exosomes	Studies involving engineered EVs were excluded	Described general mechanisms of exosome-mediated bone repair through bioactive cargo delivery, including regulation of osteogenesis, apoptosis, angiogenesis, and immune responses
95	Zhang, 2025	Systematic review and meta-analysis	10	BMSC-derived EVs; EV subtypes not systematically compared	Not systematically analyzed	Discussed potential mechanisms involving BMSC-EV-mediated regulation of osteogenesis, osteoclastogenesis, angiogenesis, and immune modulation
96	Zhang, 2025	Systematic review and meta-analysis	17	MSC-derived EVs; EV subtypes not systematically compared	Not systematically analyzed	Discussed potential mechanisms involving EV cargo-mediated regulation of osteogenesis, osteoclastogenesis, angiogenesis, and bone remodeling, with involvement of pathways such as Wnt/β-catenin, MAPK, and NF-κB pathways
97	Shuang,	Systematic	21	Stem cell-derived	Not systematically	Discussed potential mechanisms

	2025	review and meta-analysis		EVs; EV subtypes not systematically compared	analyzed	involving regulation of osteogenesis, osteoclastogenesis, and bone homeostasis through EV-associated cargo and signaling pathways, including Wnt/β-catenin, MAPK, PI3K/Akt, Hippo, and Smad pathways
Current review	Yang, 2026	Systematic review	64	Stem cell-derived EVs; EV subtypes (including exosomes, microvesicles and apoptotic vesicles) were systematically summarized	Systematic analysis of endogenous, exogenous, and integrated EV engineering strategies	Comprehensive analysis of EV-associated molecular cargo, including proteins, mRNAs, miRNAs, lncRNAs, circRNAs, and lipids, and their roles in regulating osteogenesis, osteoclastogenesis, angiogenesis, immunomodulation, and cellular senescence through multiple signaling pathways
